# Differential regulation of miRNAs involved in the susceptible and resistance responses of wheat cultivars to wheat streak mosaic virus and Triticum mosaic virus

**DOI:** 10.1186/s12864-024-10128-1

**Published:** 2024-02-28

**Authors:** Inanc Soylu, Dilip K. Lakshman, Satyanarayana Tatineni, Leny C. Galvez, Amitava Mitra

**Affiliations:** 1 Department of Plant Pathology, University of Nebraska, Lincoln, NE USA; 2 USDA-ARS Sustainable Agricultural Systems Laboratory, Beltsville, MD USA; 3 USDA-ARS Wheat, Sorghum, and Forage Research Unit, University of Nebraska, Lincoln, NE USA

**Keywords:** Wheat streak mosaic virus, Triticum mosaic virus, miRNA, Differential expression analysis, Mace, Arapahoe, Temperature-dependent expression

## Abstract

**Background:**

Wheat streak mosaic virus (WSMV) and Triticum mosaic virus (TriMV) are components of the wheat streak mosaic virus disease complex in the Great Plains region of the U.S.A. and elsewhere. Co-infection of wheat with WSMV and TriMV causes synergistic interaction with more severe disease symptoms compared to single infections. Plants are equipped with multiple antiviral mechanisms, of which regulation of microRNAs (miRNAs) is a potentially effective constituent. In this investigation, we have analyzed the total and relative expression of miRNA transcriptome in two wheat cultivars, Arapahoe (susceptible) and Mace (temperature-sensitive-resistant), that were mock-inoculated or inoculated with WSMV, TriMV, or both at 18 °C and 27 °C.

**Results:**

Our results showed that the most abundant miRNA family among all the treatments was miRNA166, followed by 159a and 168a, although the order of the latter two changed depending on the infections. When comparing infected and control groups, twenty miRNAs showed significant upregulation, while eight miRNAs were significantly downregulated. Among them, miRNAs 9670-3p, 397-5p, and 5384-3p exhibited the most significant upregulation, whereas miRNAs 319, 9773, and 9774 were the most downregulated. The comparison of infection versus the control group for the cultivar Mace showed temperature-dependent regulation of these miRNAs. The principal component analysis confirmed that less abundant miRNAs among differentially expressed miRNAs were strongly correlated with the inoculated symptomatic wheat cultivars. Notably, miRNAs 397-5p, 398, and 9670-3p were upregulated in response to WSMV and TriMV infections, an observation not yet reported in this context. The significant upregulation of these three miRNAs was further confirmed with RT-qPCR analysis; in general, the RT-qPCR results were in agreement with our computational analysis. Target prediction analysis showed that the miRNAs standing out in our analysis targeted genes involved in defense response and regulation of transcription.

**Conclusion:**

Investigation into the roles of these miRNAs and their corresponding targets holds promise for advancing our understanding of the mechanisms of virus infection and possible manipulation of these factors for developing durable virus resistance in crop plants.

**Supplementary Information:**

The online version contains supplementary material available at 10.1186/s12864-024-10128-1.

## Introduction

Wheat streak mosaic virus (WSMV) and Triticum mosaic virus (TriMV) are two common viruses causing foliar diseases of wheat in the Great Plains region of the U.S.A. WSMV is a monopartite, positive‐sense, single‐stranded RNA virus and the type member of the genus *Tritimovirus* in the family *Potyviridae*. WSMV is transmitted by the wheat curl mite (*Aceria tosichella* Keifer). TriMV is the type species of the genus *Poacevirus* in the family *Potyviridae.* It has genomic organization similar to WSMV and is also transmitted by the wheat curl mite [1–3]. While these two viruses can cause a total crop loss, typical annual yield losses range from 3 to 5%, with an estimated $76 million in Kansas state alone [4]. Co-infection of plants with WSMV and TriMV causes synergistic interaction with more severe disease symptoms [1, 5]. Plants are equipped with multiple antiviral mechanisms, silencing of viral RNA induced by small RNAs is an evolutionary conserved and perhaps most effective against RNA viruses. Host resistance genes are also a major source of resistance against plant viral diseases. Genetic resistance against WSMV, TriMV, or both have been used by deploying non-allelic *Wsm1* or *Wsm2* into commercial wheat cultivars [6, 7]. Wheat cultivars with *Wsm1* or *Wsm2* gene provide resistance to WSMV plus TriMV and WSMV, respectively, at or below 18 °C by preventing virus entry into the vasculature, and resistance is overcome at higher temperatures [8–10]. The third gene, *Wsm3*, provides resistance against both WSMV and TriMV and has been shown to be effective at higher temperatures [11].

As obligate parasites, plant viruses depend on hosts to successfully complete their life cycles by co-opting many host proteins and utilizing host mechanisms [12]. It has been well-established that many aspects of plant growth, development, and environmental response are controlled by small RNAs (sRNAs) [13]. Plant sRNAs are classified into two major classes: microRNAs (miRNAs) and small interfering RNAs (siRNAs). RNA modulation induced by sRNAs is a central regulator of gene expression and an evolutionarily conserved mechanism in eukaryotic organisms [14]. Since the discovery of gene silencing in plants, siRNAs have been intensively studied for defense against virus infections [15]. microRNAs are a class of small, endogenous RNAs that are involved in regulating post-transcriptional gene expression. The primary distinction between siRNAs and miRNAs is that the latter regulate the expression of several mRNAs while the former suppress the production of a single target mRNA, mostly their own loci. In contrast, most miRNAs silence other genes [16, 17]. Plant miRNAs, along with a vital role in numerous regulatory pathways involving growth and development, are also involved in plant-virus interactions [18]. There are some host miRNAs that function as proviral miRNAs helping viruses invade and flourish within the plant tissues [19, 20]. Similarly, plants also use miRNAs to target virus genomes for degradation or suppression of translation and replication [19, 20]. However, the complex dynamics of miRNA influencing virus infection are largely unknown.

We previously studied the synergistic interaction between WSMV and TriMV on endogenous and virus-derived small interfering RNAs by examining susceptible wheat cultivar (cv.) Arapahoe and temperature-sensitive resistant wheat cv. Mace at 18 °C and 27 °C [21]. WSMV and TriMV interact synergistically in co-infected wheat cultivar Arapahoe at 18 °C and 27 °C, and in Mace only at 27 °C but not at 18 °C. We found that wheat cv. Arapahoe infected by WSMV and TriMV at both 18 °C and 27 °C and wheat cv. Mace infected by WSMV or TriMV at 27 °C caused a drastic reduction in the accumulation of 24 nt endogenous sRNAs compared to healthy wheat cultivars. Co-infection of Arapahoe at both temperatures and Mace at 27 °C caused a significant shift in sRNA accumulation compared to healthy wheat cultivars. In an effort to elucidate the role of miRNA in wheat-WSMV/TriMV interactions, we employed bioinformatics tools and reverse transcription-quantitative polymerase chain reaction (RT-qPCR) to study and validate the dynamics of host miRNA in single and double infections in both the wheat cultivars, at permissive and non-permissive temperatures.

In this study, our central approach revolved around the application of computational methods to investigate the interactions between wheat miRNA transcriptome and TriMV or WSMV, as well as their combined infections. This approach conferred several distinct advantages. First, we were able to scan the entire known miRNA transcriptome to discern changes elicited by infections. This led us to further confirm some of the responders induced by the infections, such as miRNA 319 [22], miRNA 397 [23], and miRNA 398 [24], while also identifying novel functions for already known miRNAs like, miRNA 9670 and miRNA 5384. A secondary advantage stemming from our computational approach lies in its capacity to incorporate even the least abundant miRNAs within our analyses – an endeavor that is often challenging through traditional experimental routes.

## Results

### Perturbations occur in conserved miRNAs in response to TriMV and WSMV infections

We mapped miRNA reads obtained in our previous study [21] to a reference database of mature miRNA sequences from wheat. The sequence reads were from the samples of two wheat cultivars, Arapahoe and Mace which were subjected to different viral treatments: TriMV, WSMV, and the double infection of both the viruses, as well as mock-inoculated control samples at 18 °C and 27 °C. By mapping the sequence reads to known mature miRNA sequences, we generated sixteen count tables (of miRNA abundance), one for each case. A total of 101 miRNAs, out of 147, were represented in the count tables after noise removal and normalization. All nine of the highly conserved miRNA families [25] miR156, miR159, miR160, miR166, miR168, miR171, miR172, miR390, and miR396 had presence in our datasets. When miRNA abundance was evaluated independently of viral treatments, the most abundant miRNA family for both Arapahoe and Mace was miR166. The abundance of miR166 was 282,703 at 18 °C and 566,592 reads at 27 °C for wheat cv. Arapahoe; while for cv. Mace, with 277,618 at 18 °C and 151,669 at 27 °C. The miRNA166 abundance was followed by miRNA 159a and miR168a in both cultivars.

**Fig. 1:**
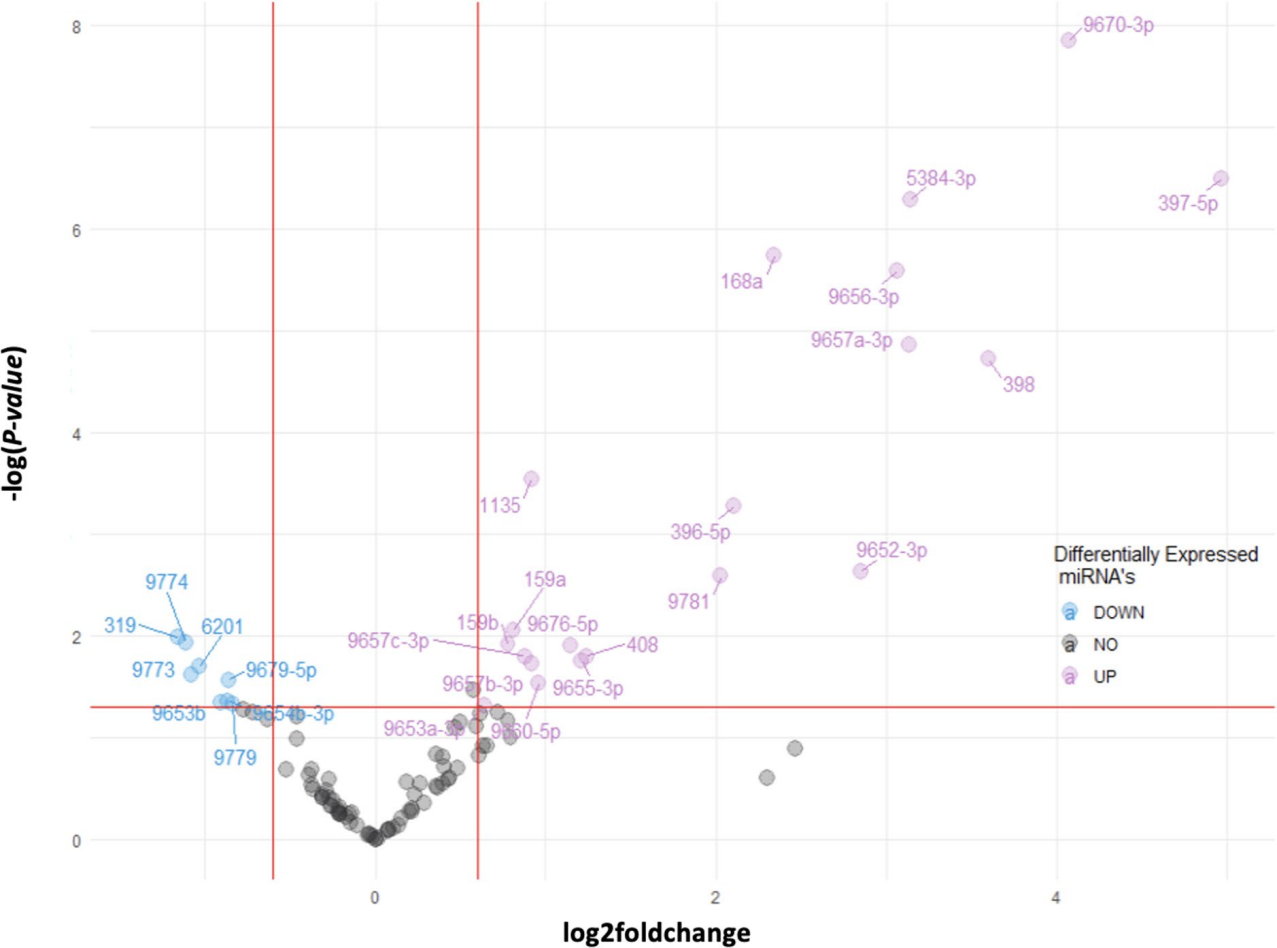
Differentially expressed miRNAs of the infection group. Red lines separate significantly (*p*-value < 0.05) up regulated (log2foldchange > 0.6) and down regulated (log2foldchange < -0.6) miRNAs. Less abundant miRNAs 9670-3p, 397-5p, and 5384-3p are strongly associated with the infection groups

Before delving into in-depth analysis, we initially explored the effects of viral infections (i.e., WSMV, TriMV, and double infection of both viruses) on miRNA abundance. For this purpose, we conducted a differential expression analysis on mock- and virus-inoculated groups. Figure 1 shows the differentially expressed miRNAs in the virus-inoculated samples. Overall, the expression of 28 miRNAs was significantly affected following the infection (*p*-value < 0.05). Out of these 28, 20 miRNAs were upregulated (log2foldchange > 0.6), while 8 miRNAs were downregulated (log2foldchange < -0.6). Notably, miRNAs 9670-3p, 397-5p, and 5384-3p exhibited the most significant upregulation, whereas miRNAs 319, 9773, and 9774 were the most downregulated ones.

**Fig. 2:**
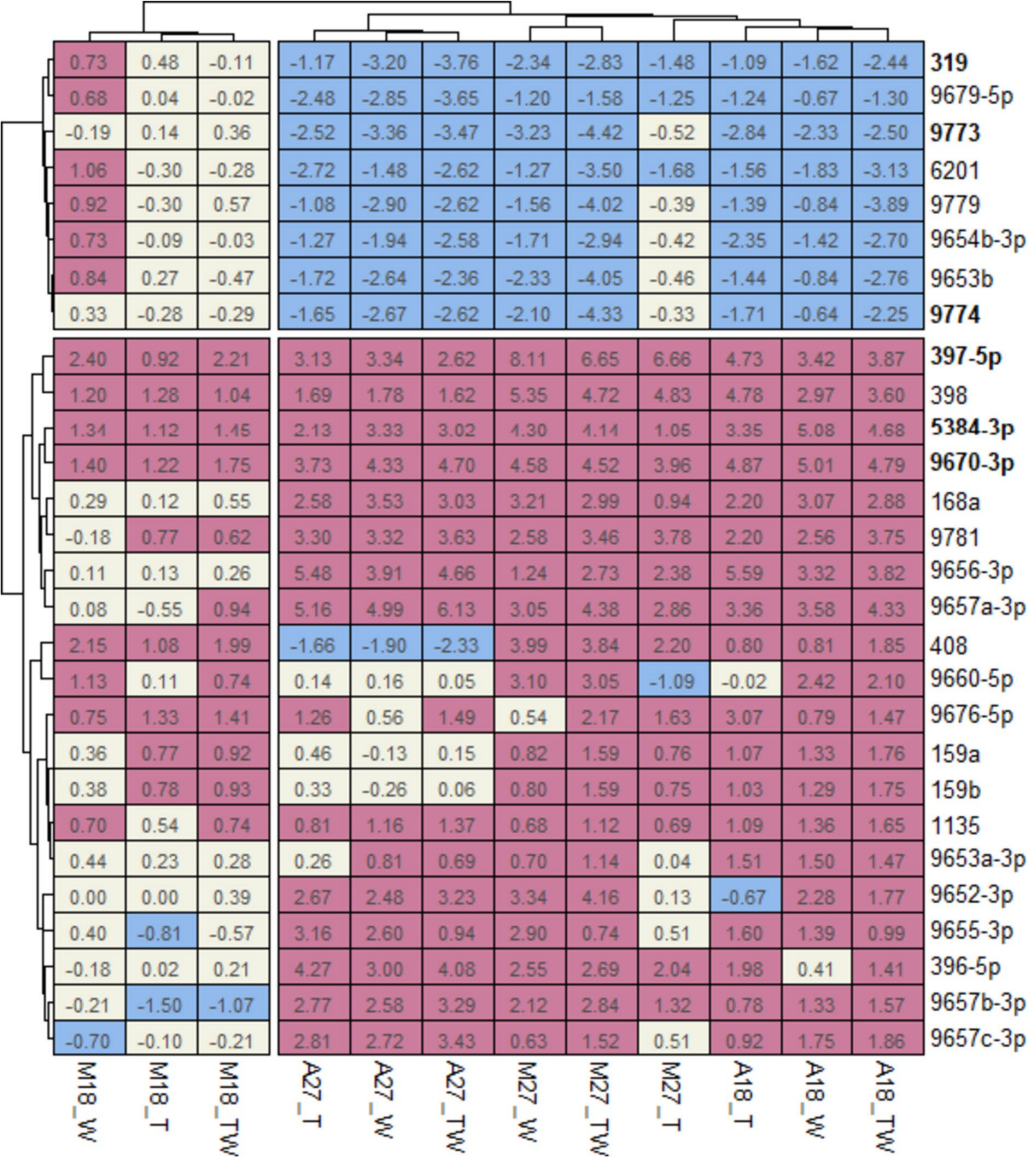
Heatmap of the 28 miRNAs identified in the DGE analysis. Each box in the heatmap represents the log2foldchange value for a given miRNA (row) inoculated with a virus (column) compared to the control (mock-inoculated) at given temperature. Twenty upregulated miRNAs were clustered below the vertical divide, while eight downregulated ones were clustered above. Viral treatments of Mace at 18 ºC was separated from the other infected groups with the horizontal divide. T: TriMV, W: WSMV, and TW: double-infection. M: Mace and A: Arapahoe. 18: at 18 ºC, 27: at 27 ºC. Red indicates upregulated miRNAs, while blue indicates downregulated miRNAs

Next, we investigated whether wheat cultivar or temperature has any effect on the changes in miRNA expression induced by viral infection. Figure 2 shows the heatmap of the 28 miRNA identified by the DGE (Fig. 1). Each value in the heatmap represents the log2foldchange for a sample treated with virus/es in comparison to the mock-inoculated sample under the same temperature. Among these, the abundance of miRNA 9670-3p (Fig. 2, with bold) increased by 4.70- and 4.79 -fold due to double infection in wheat cv. Arapahoe at 27 ºC and 18 ºC, respectively. Notably, in wheat cv. Mace while no significant changes were observed at 18 ºC, at 27 ºC the expression of the same miRNA increased by 4.52-fold. The same trend was observed for miRNA 5384-3p; there was an increase of 3.02- and 4.68-fold for Arapahoe at 27 ºC and 18 ºC, respectively. While no significant change was observed for resistant cv. Mace at 18 ºC, the abundance of miRNA 5384-3p increased by 4.14-fold for Mace at permissible temperature 27 ºC. For miRNA 397-5p, irrespective of the cultivar and temperature, double infection consistently increased its abundance.

The abundance of certain miRNAs abated due to the viral infection. One such example is miRNA 319, which exhibited reduced abundance in Arapahoe at 18ºC and 27ºC, and in Mace at 27ºC by 2.44-, 3.76-, and 2.83-fold, respectively. However, there was no significant change observed in the abundance of this miRNA for Mace at 18ºC (0.11) which was resistant to WSMV and TriMV at 18 ºC. A similar trend was observed for miRNA 9773 in Arapahoe and Mace at 18ºC and 27ºC by 2.50-, 3.47-, 0.36-, and 4.42-fold respectively, and also for miRNA 9774 (2.25-, 2.62-, 0.29, and 4.33-fold). The miRNA abundance and fold expression changes are available as Supplementary information (Tables S1-S2).

**Fig. 3:**
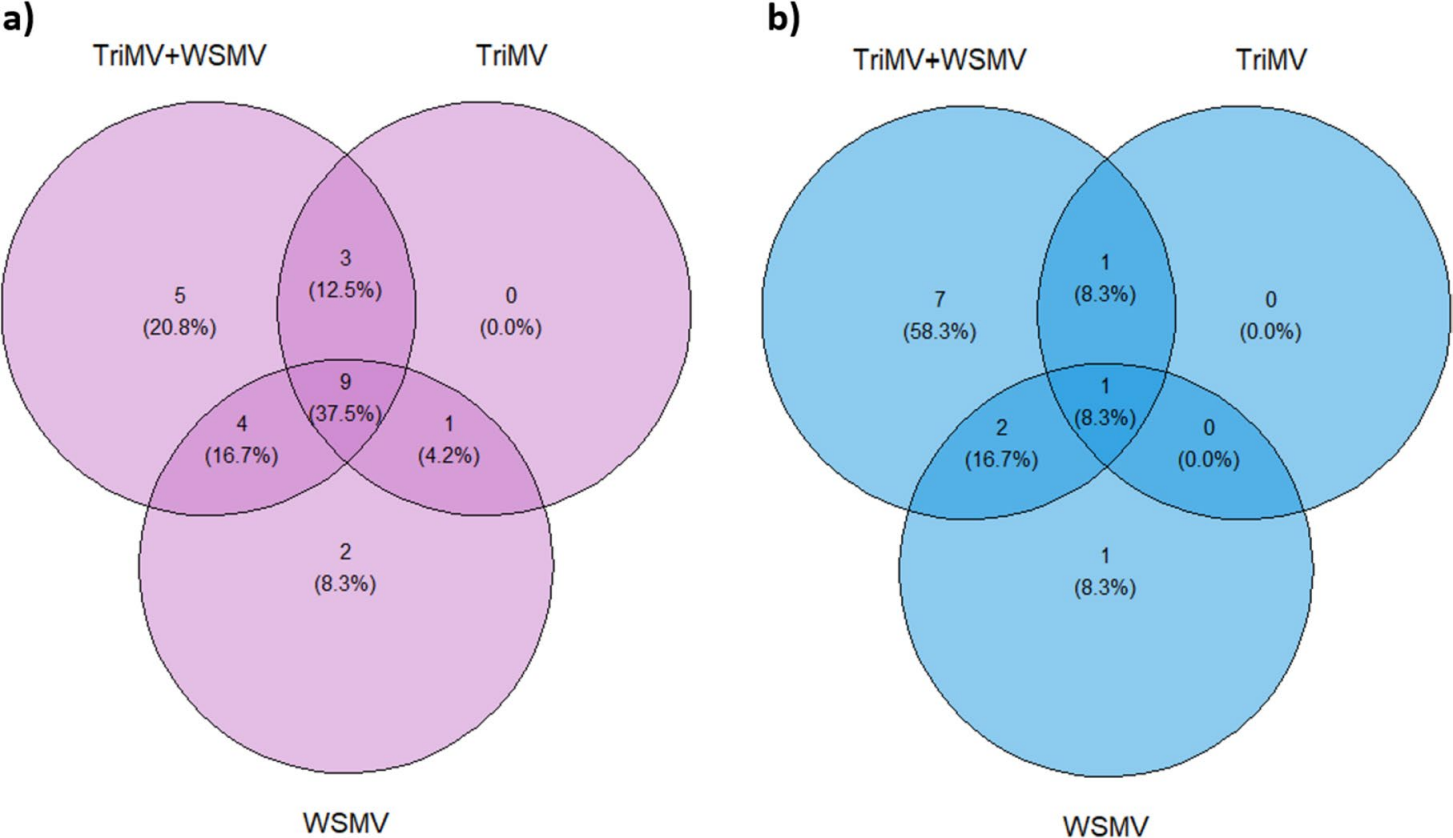
Venn diagram of differentially expressed miRNAs. Diagrams illustrate **a**) upregulated miRNAs, and **b**) downregulated miRNAs in singly- (TriMV or WSMV) or doubly (WSMV + TriMV) infected wheat samples compared to mock-inoculated samples

Expanding on this pairwise comparison (infected vs control), we then determined differentially expressed miRNAs for each infection. Across all infections, nine miRNAs were consistently upregulated (Fig. 3a), and one miRNA was found to be downregulated (Fig. 3b). In the case of double infection and WSMV, miRNAs 9670-3p, 397-5p, and 5384-3p (which are the most significantly upregulated miRNAs, as shown in Fig. 1) were among the most upregulated miRNAs. However, in the case of TriMV, the change in miRNA 397-5p and miRNA 5384-3p expression was not as striking as in the other two infection groups (Table S3). A similar tendency was observed for the most downregulated miRNAs: 319, 9773, and 9774. While they were among the downregulated miRNAs in the double infection and WSMV group, they were absent for the TriMV group. A mutually downregulated miRNA for all infection groups was miRNA1120a.

The miRNAs identified through the previous approaches (the holistic approach of Fig. 1 and the infection-based approach of Figs. 2 and 3) helped us identify certain miRNAs as responders to the infections of TriMV and WSMV. However, investigating their abundance across different varieties, temperatures, and infections requires a distinct approach. This is particularly crucial since the wheat cv. Arapahoe is susceptible to TriMV and WSMV infections at 18ºC and 27ºC, while cv. Mace is only susceptible at 27ºC but resistant at 18ºC [21]. To explore whether the variations in the numerous miRNAs caused by viral infections can be summarized and visualized, we employed Principal Component Analysis (PCA). Additionally, our aim was to identify the miRNAs that contribute the most to the aforementioned observations.

### Less abundant miRNAs are strongly correlated with the symptomatic infections in wheat cultivars

Before conducting a PCA, we assessed the suitability of our datasets for this analysis by employing the Kaiser–Meyer–Olkin (KMO) test and Bartlett's Test of Sphericity (BTS). The KMO test evaluates the adequacy of the provided factors, and our datasets scored 0.92 (> 0.7 is considered good for PCA [26]). The BTS, which determines whether a matrix significantly differs from an identity matrix, yielded a p-value lower than 0.05. Taken together, these results led us to the conclusion that our datasets were well-suited for PCA. Five principal components were extracted from our miRNA dataset, each with eigenvalues greater than 1. These components cumulatively accounted for 84.42% of the total variance (Table 1).

**Table 1:** Loading matrix derived from principal component analysis of wheat miRNAs. Top 6 miRNAs associated with symptomatic samples

	**PC1**	**PC2**	**PC3**	**PC4**	**PC5**
Eigenvalue	68.324	31.407	19.442	9.264	8.517
Variability (%)	42.115	19.360	11.984	5.711	5.250
Cumulative (%)	42.115	61.475	73.459	79.170	84.420
Loadings					
397-5p	2.767	0.434	-0.085	-0.456	0.510
9670-3p	2.412	-0.134	-0.045	-0.122	0.021
9657a-3p	2.022	0.443	-0.331	0.091	-0.391
398	1.651	1.154	-0.533	-0.339	0.183
5384-3p	1.599	-0.317	-0.348	0.503	0.282
9656-3p	1.571	-0.264	0.070	0.062	-0.454

**Fig. 4:**
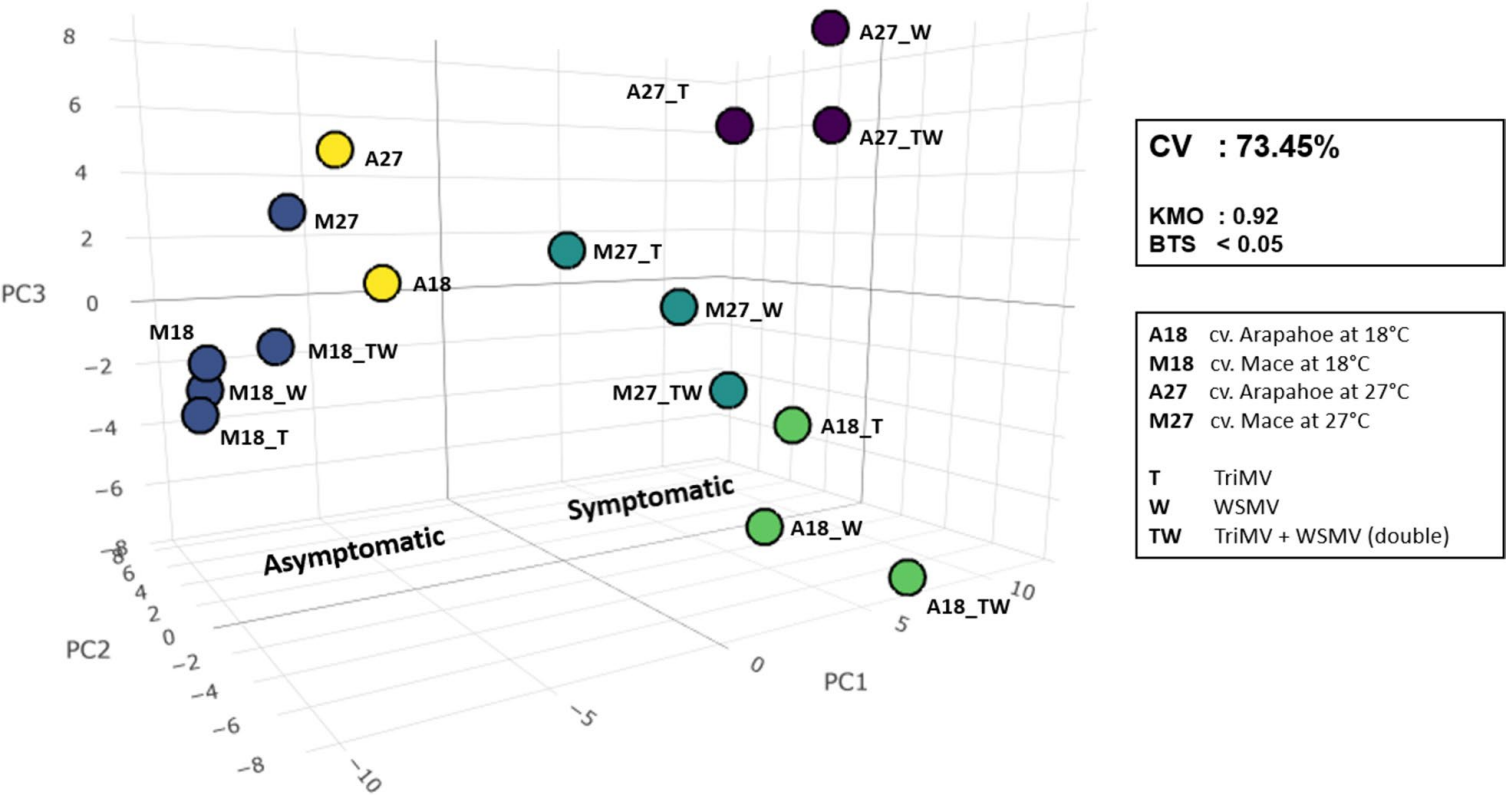
Three-dimensional scatter plot for the first three principal components that were analyzed using the principal component analysis (PCA) for wheat miRNAs. Colors assigned to each point represents their respective cluster determined by K-means algorithm. CV: Cumulative variance, KMO: Kaiser–Meyer–Olkin criterion, BTS: Bartlett’s test of sphericity

To interpret this data while preserving the maximum amount of information (73.46%), we plotted the first three principal components in a three-dimensional space (Fig. 4). These principal components explained 42.12%, 19.36%, and 11.98% of the total variance, respectively. For clustering the data points, we employed the K-means clustering algorithm and determined the optimal number of clusters using the silhouette method. By plotting the average silhouette width against the number of clusters (Figure S1) revealed a distinct peak at five clusters. Additionally, the same graph exhibited two additional local maxima at two and seven clusters.

With five clusters, we observed three well-differentiated clusters that successfully segregated symptomatic infected groups: the virus-infected Arapahoe at 18 °C (green cluster), Arapahoe at 27 °C (purple cluster), and Mace at 27 °C (teal cluster). The remaining two clusters corresponded to asymptomatic or mock-inoculated groups of Arapahoe (yellow cluster) and Mace (blue cluster) at two different temperatures. Notably, in the case of Mace at 18 °C, the virus-infected groups were clustered together with the control mock-inoculated groups. Another biologically important separation was observed with two clusters, effectively dividing the data space into symptomatic (virus-inoculated and asymptomatic (uninfected or mock-inoculated) groups.

**Fig. 5:**
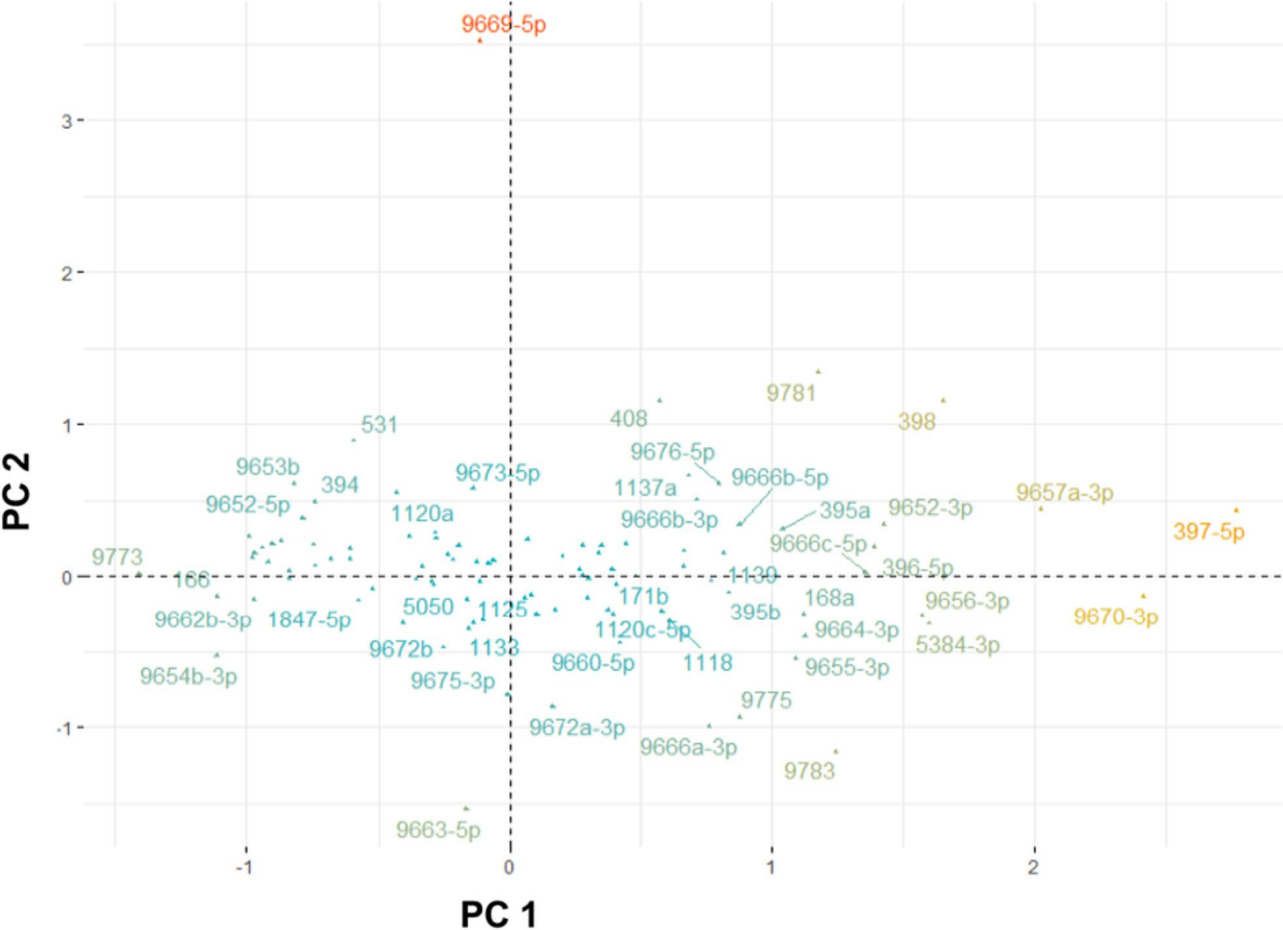
Loadings for the first two principal components; PC1 and PC2. Color code (i.e., blue from red) applied to indicate the most contributing miRNA

Figure 5 shows the loadings for the first two principal components. When considered alongside Fig. 4, several less abundant miRNAs demonstrated positive loadings on PC1, indicating a strong correlation with the symptomatic groups. Specifically, miRNAs 397-5p, 398, 9656-3p, 5384-3p, 9657a-3p, and 9670-3p, which exhibited lower abundance in the mock-inoculated samples (Table S1), displayed a strong and positive association with the symptomatic groups (Fig. 5 and Table 1). In contrast, miRNAs 9773, 9654b-3p, and 166 exhibited negative loadings on PC1, suggesting that they are downregulated with the infection. The miRNAs loaded onto the second principal component contributed to the cultivar difference, distinguishing Arapahoe from Mace. miRNAs 9669-5p and 9663-5p were the most significant contributors to the PC2 loadings.

### miRNAs 397-5p, 398, and 9670-3p are upregulated in response to virus infection

The principal component analysis revealed a positive association, in decreasing order, between miRNAs 397-5p, 9670-3p, 9657a-3p, 398, and 9656-3p, and symptomatic viral infections. To experimentally validate these findings, we selected the top three miRNA contributors for the symptomatic groups and performed RT-qPCR to assess their increased presence in total RNA. Given their comparable contributions in loadings and the low copy count of miRNA 9657a-3p in the total RNA (Table S1), we chose to use miRNA 398 instead of miRNA 9657a-3p for technical convenience. 5' primers were designed by utilizing the mature miRNA sequences and were paired with a generic reverse primer, pTR, in the RT-qPCR assay (Table S4). We also designed primers to test miRNAs 5384-3p and 9773, which stood out in previous analysis (Figs. 1 and 2), but did not perform well (i.e., amplified multiple targets; data not shown), most likely due to their repetitive and low complex sequences.

To further mitigate the coincidental elevation of the selected miRNAs, we conducted a new set of infections identical to our previous study [21] and utilized them in our RT-qPCR experiments.

**Fig. 6:**
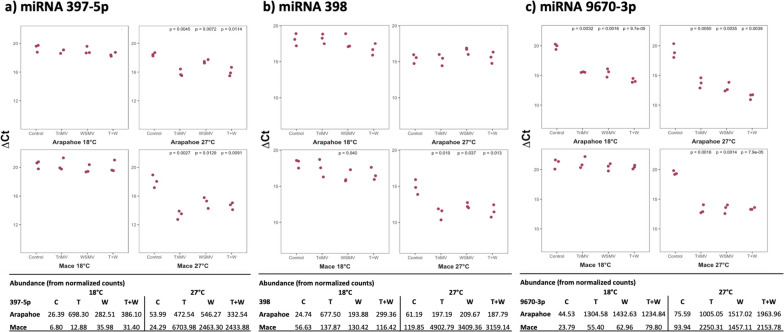
One-dimensional scatter plots of three selected miRNA expression (ΔCt) in mock and virus inoculated leaves of wheat cultivars Arapahoe and Mace at 18* °C* and 27* °C*. A lower ΔCt value represents higher expression of **a**) miRNA 397-5p, **b**) miRNA 398, and **c**) miRNA 9670-3p. Abundance from normalized counts is provided below each graph to provide comparison between RNAseq data and miRNAs expression levels obtained in RT-qPCR. Each dot represents the ΔCt value of a biological replicate. *P*-values for statistically significant samples are depicted above each sample. T: TriMV, W: WSMV, T + W: both viruses

Figure 6 shows the gene expression (ΔCt) in inoculated samples (mock- and virus-) of wheat cultivars Arapahoe and Mace at 18 °C and 27 °C. A lower ΔCt indicates a more abundant miRNA in the sample, and thus, higher expression. The expression of miRNA 397-5p (Fig. 6a) exhibited a significant increase with all infections in both Arapahoe and Mace at 27 °C. However, under different viral treatments, we observed no meaningful increase in the expression of miRNA397-5p for Mace and Arapahoe at 18 °C. The levels of miRNA398 (Fig. 6b) were elevated in the case of TriMV, WSMV, and double infection for Mace at 27 °C. Additionally, for Mace at 18 °C with WSMV, we observed a slight increase in expression. MicroRNA 9670-3p (Fig. 6c) showed significant increase in expression in both cultivars at 27 °C with all infections. Moreover, an increase in expression was observed for the same miRNA with TriMV, WSMV, and double infection for Arapahoe at 18 °C. However, for Mace at 18 °C, we did not detect an increase in miRNA 9670-3p expression. Our miRNA9670-3p results were perfectly in line with RNAseq data. The dissociation curves (Fig. S2) plotted for each miRNA:pTR primer pair exhibited single peaks, confirming the accurate amplification of the chosen miRNA within the assay.

### Predicted targets of selected miRNAs

Our validation experiments have revealed less abundant miRNAs are affected by symptomatic infections of TriMV and WSMV. Specifically, we have chosen to focus on a select miRNA—miRNAs 319, 397-5p, 398, 5384-3p, 9670-3p, 9773, and 9774—that exhibit a connection to viral infections. Following stringent filtering criteria, we have identified a total of 148 potential targets for these selected miRNAs (Table S5). The number of potential targets associated with each miRNA ranged from 2 to 92. Notably, miR5384-p stands out with the highest number of potential targets, namely 96 genes, followed by miR319 which targeted 18 genes. The remaining miRNAs each target between 2 and 10 genes.

**Fig. 7:**
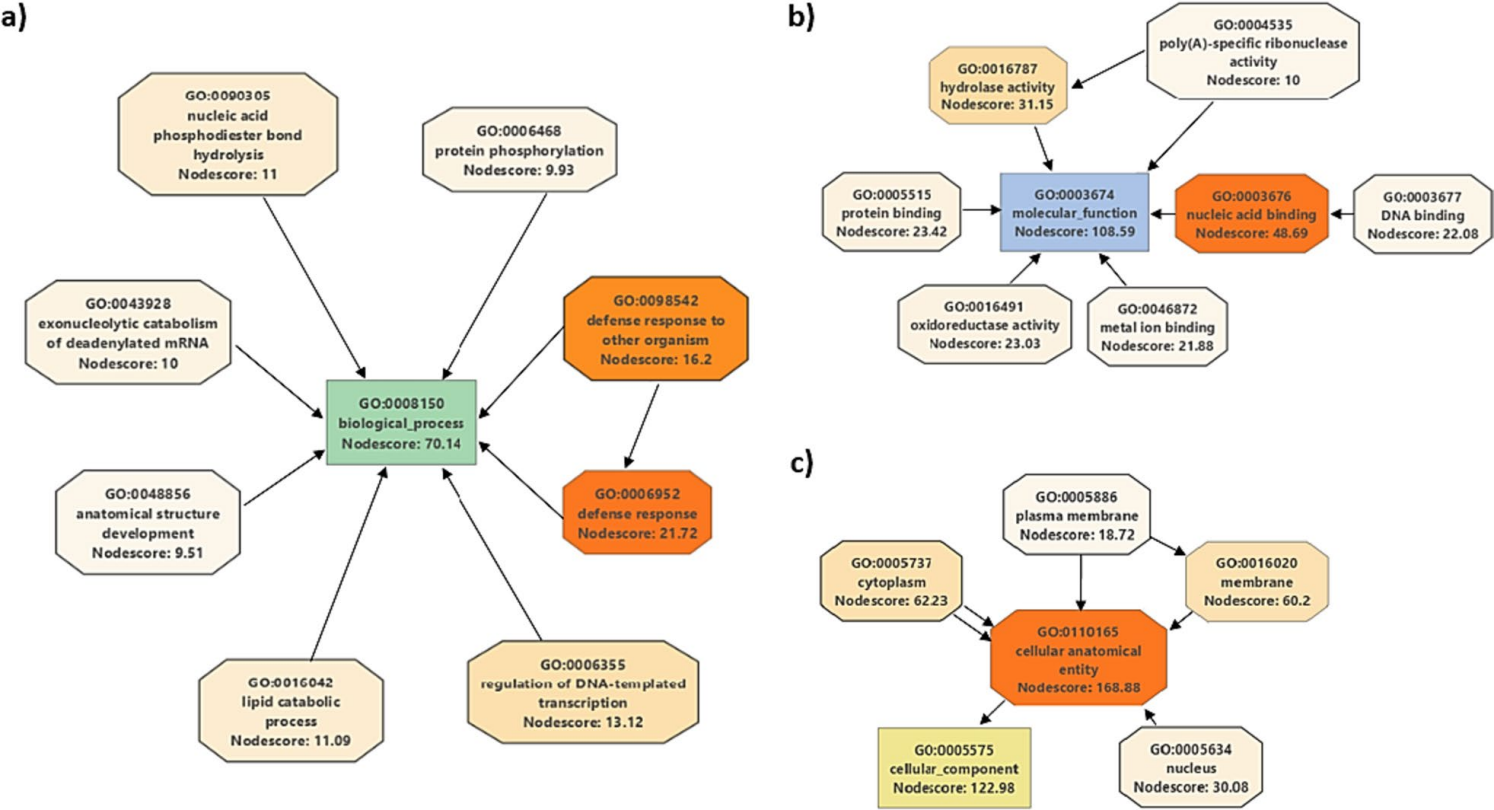
Gene Ontology (GO) annotation diagrams. The targets were annotated in three main categories: **a**) biological process (green anchor), **b**) molecular function (blue anchor), and **c**) cellular component (orange anchor). The nodes are color-coded on a gradient scale, ranging from white to orange, based on their nodescore

To understand the nature of these targets, we executed a BLAST search against the non-redundant protein database, subsequently carrying out annotation through utilizing both the Gene Ontology (GO) and InterPro databases. With the exception of 25 targets, all other targets were successfully annotated with their corresponding GO terms. The simplified diagrams (i.e., with all the intermediate functions filtered out, Fig. 7) shows the annotation of targets across the three major GO categories: biological process, molecular functions, and cellular component. These targets are involved in diverse spectrum of biological process, with a peculiar finding concerning their intricate association with defense responses to other organisms (GO:0006952 and GO:0098542). Moreover, they were implicated in processes such as regulation of DNA-templated transcription (GO:0006355), lipid catabolic process (GO:0016042) and nucleic acid phosphodiester bond hydrolysis (GO:0090305) (Fig. 7a). The primary molecular functions observed among these targets are nucleic acid binding (GO:0003676), coupled with various catalytic activities. Significantly, the most commonly shared molecular functions within the latter category are hydrolase (GO:0016787) and oxidoreductase (GO:0016491) activities (Fig. 7b). Finally, as for their cellular localization, most of the target genes are categorized under the term “cellular anatomical entity”, with comparable localization in both the membrane and cytoplasm. A minority of these genes is situated within the nucleus (Fig. 7c).

## Discussion

In this study, we investigated the changes in miRNA abundance in susceptible (cv. Arapahoe) and temperature-sensitive resistant (cv. Mace) wheat cultivars that were singly- or doubly-inoculated with TriMV and WSMV at two different temperatures. Our aim was to examine the involvement of different miRNAs in disease development by WSMV and TriMV in wheat. First, we performed a differential gene expression analysis (DGE). Our analysis showed (Fig. 1) that 28 miRNAs were differentially expressed in wheat inoculated with WSMV and TriMV. Twenty of these miRNAs were significantly upregulated (*p*-value < 0.05, log2foldchange > 0.6), among them miRNAs 168a, 397-5p, 398, 5384-3p, and 9670-3p were the most significant ones. Virus mediated miRNA 168 induction, as in our case, has been previously linked to suppression of endogenous AGO1 level and thus causing susceptibility [27]. Similarly, overexpression studies linked these miRNAs to susceptibility against certain diseases, like overexpression of miRNA 397 to powdery mildew in wheat [28] and miRNA 398 to verticillium wilt in cotton [29]. Although there are studies showing these miRNAs can also be downregulated in certain plant-pathogen systems [30, 31]. Regardless of direction, it is important to note that the differentially expressed miRNAs are linked to susceptibility to diseases. For miRNAs 5384-3p and 9670-3p, our study is the first to report their involvement in the progression of WSMV and TriMV infections in wheat. Some miRNAs were significantly downregulated (*p*-value < 0.05, log2foldchange < 0.6) in response to viral treatments. One such example is miRNA 319, which is also known for its involvement in plant pathogen response [32], along with it we identified miRNAs 9773 and 9774 are also involved in WSMV and TriMV infections.

Our DGE analysis primarily focused on the infection. As a result, it was somewhat limited in its ability to effectively convey our temperature-dependent data. To address this limitation, we utilized the normalized counts obtained from our DGE analysis. We then proceeded to compare the samples with viral treatments against mock samples at various temperatures (Fig. 2). Special attention was given to the miRNAs that stood out in our DGE analysis (i.e., the most significant ones, *p*-value < 0.05). This approach revealed an intriguing trend that began to take shape. For instance, in the case of miRNA 319, at 18ºC, Arapahoe with double infection exhibited a significant downregulation, whereas Mace with double infection demonstrated a similar expression level to the control group. However, at 27ºC, the latter also exhibited a significant downregulation. Similarly, miRNAs 397-5p, 398, 5384-5p, 9656-3p, 9670-3p, 9773, and 9774 exhibited the same trend. Considering that Arapahoe is susceptible to double infection at both temperatures, whereas Mace is only susceptible at 27 °C, the differential expression of these miRNAs are likely to be associated with the infections. Not all differentially expressed miRNAs were common across all treatments (Fig. 3). While certain miRNAs were shared among all treatments, such as miRNAs 168, 397, 398, 5384-3p, and 9670-3p, some miRNAs were only differentially expressed in certain single virus treatments. For example, miRNA 319 was downregulated for WSMV and double infection treatments, whereas miRNA 9781 upregulated in TriMV and double infection treatments. Likewise, in case of miRNA 9656-3p, TriMV induced a higher expression than WSMV, as outcome in double infections results expression levels that are more closely aligned with the former, i.e., TriMV than WSMV (see Supp. Table S3). This interplay of differential expression induced by two distinct plant viruses could render the plant even more susceptible state, potentially leading to a more severe infection due to the synergistic effect [21, 33].

The idea of an interplay among several miRNAs into a more severe disease prognosis motivated us to perform PCA on all treatments, incorporating all miRNAs simultaneously (Fig. 4). To further enrich our analysis, we applied the K-means algorithm to cluster these points. Five clusters were formed following the clustering algorithm. Notably, each cluster had members only from the same cultivar pointing to, as expected, intra-cultivar miRNA expression was similar for both Arapahoe and Mace.

Interestingly, for Arapahoe, the mock-inoculated control group (yellow) and viral treatments at 18 °C and 27 °C formed distinct clusters. Firstly, control groups at different temperatures clustered together (yellow), suggesting that the temperature’s effect on miRNA expression was limited compared to viral treatments. Secondly, in the case of Arapahoe’s viral treatments, temperature played a more crucial role. Despite different viral treatments, each treatment clustered together for a specific temperature. This indicates that viral treatments have varying effects on miRNA composition at different temperatures, showing different profiles compared to the mock group (yellow cluster). A similar pattern was observed for the Mace cultivar at 27 °C. Here, control groups congregated within the blue cluster, while viral treatments at 27 °C clustered in the teal cluster. However, a notable separation observed for the viral treatments of Mace at 18 °C, which formed a cluster alongside the mock-inoculated control group. This indicates that viral treatments lacked the ability to alter the miRNA profile of Mace at 18 °C, perhaps explaining why Mace is resistant to these infections at lower temperatures.

As an important finding in our PCA, PC1 (i.e., X-axis in Fig. 4) was positively and strongly associated with the viral treatments. Notably, Mace at 18 °C, being failed to be infected, was clustered with the mock-inoculated group, indicating miRNAs loaded on PC1 were specific to the disease progression. At first glance, miRNA 397-5p, 398, 5384-3p, 9670-3p, and 9657a-3p had strong contributions to symptomatic virus-inoculated groups. Conversely, miRNA 9773 had a strong contribution to the mock-inoculated asymptomatic groups, signifying its downregulation in samples subjected to viral treatment. These miRNAs consistently emerged in all of our analyses. However, these miRNAs exhibited low count (< 100) in healthy samples, thus potentially rendering them unreliable. To address this issue, and experimentally proving their existence (specifically for miRNA 9670-3p), we tried to quantify the miRNAs 397-5p, 398, 5384-3p, 9670-3p, and 9773 by RT-qPCR. The miRNAs 5384-3p and 9773 did not perform well, possibly due to low complexity. However, for miRNAs 397-5p, 398, and 9670-3p, our RT-qPCR results was mostly in agreement with our computational data (Fig. 6). Both miRNAs 397-5p and 9670-3p showed elevated expression in response to TriMV, WSMV, and double infections at 27 °C for both cultivars. The latter also exhibited elevated expression in Arapahoe 18 °C in response to infections. For miRNA 398 in Mace 27 °C, all viral-inoculated samples showed increased expression. In a few cases, although bioinformatics data suggested minimal increase in abundance, our RT-qPCR experiment did not detect it. We hypothesize that the complex nature of the validation experiment (i.e., low abundant miRNAs, inefficient tailing), and different biological samples might have caused this observed difference. The same reasons could be behind the unexpected elevation of miRNA for Mace at 18 °C with WSMV infection; similar contradictory results have been reported before [34]. The involvement of miRNAs 397 and 398 were previously known in plant host- pathogen interactions, but this study is the first to associate them with TriMV and WSMV infections. Furthermore, miRNA 9670-3p, a previously known but not well-categorized miRNA has been shown to be a strong negative regulator of TriMV and WSMV infections.

miRNAs are important gene regulatory elements and due to their plasticity in complementarity they can regulate the expression of target genes in different degrees [35]. Yet the identification of their targets across the genomes is still challenging due to the very same reason. A too stringent algorithm may miss a potential target for miRNAs. Although several tools were developed to identify novel miRNAs based on a set of rules (e.g., seed sequence complementarity), they are far away from being standard. To discover potential targets for the miRNA’s that were discussed in this study we employed previously established criteria (see Methods) and identified their potential targets. For our search, we included the five most upregulated (miRNAS 397-5p, 398, 5384-3p, 9657-3p, and 9670-3p) and three most downregulated (miRNAs 319, 9773, and 9774) miRNAs. Through our searches, we found 148 (Table S5) potential targets for these miRNAs. Subsequent GO annotation revealed their involvement in three major categories: biological process, molecular function, and cellular component (Fig. 7). Broadly defined, these target genes are involved in defense response to other organism, exhibiting catalytic and nucleic acid binding activity, with a small portion of them are located in the nucleus. Individually, some of these targets align with previously reported targets. For instance, miRNA 319 targets MYB and TCP21 genes in rice [36], and its overexpression causes susceptibility to the blast disease. Similarly, according to our target analysis, tae-miRNA 319 also targets MYB-like transcription factors, which can then be employed by the virus, and it is differential expression of tae-miRNA 319 may be hypothesized to be linked to susceptibility to TriMV and WSMV. For miRNA 397, we were unable to characterize its potential targets; however from the literature we know it is involved in lignin biosynthesis, overexpression of it reduced the lignin deposition and increased susceptibility to fungal pathogens [23]. miRNA 398 targets integrator complex which has been found to regulate miRNA abundance [37], as well as superoxide dismutase which has been previously linked to several infections as both negative and positive regulator of the infection [38, 39]. miRNA 5384-3p has the most targets of all miRNAs studied in this analysis, including transcription factors, autophagy related proteins, plant hormone transduction and several catalytic enzymes. miRNA 9670-3p also targets plant hormone as well as two RGA-like disease resistance proteins, however more studies required specifically on the latter two miRNAs to further elaborate their involvement respect to TriMV and WSMV infections.

## Conclusion

In conclusion, this study demonstrates that the wheat miRNA transcriptome displays significant temperature-dependent differential expression in response to TriMV and WSMV infections. Notably, several of these miRNAs have not been previously associated with these infections. As a result, further investigation into the roles of these miRNAs and their corresponding targets holds promise for advancing our understanding of the mechanisms of virus infection. This potential avenue of research could yield valuable insights into the intricate interactions between wheat and these viral pathogens. As virus life cycles primarily rely on host factors, identification of critical host factors and understanding their role may allow us to manipulate these factors for durable virus resistance in crop plants.

## Methods

### Sequence acquisition and preparation for the downstream analysis

Small RNA library sequences [21] were downloaded from GEO Omnibus using accession codes from GSM1306034 to GSM1306049. This library has a collection of 16 sets of RNA sequences; each dataset contained the reads from a group of three individuals of a wheat cultivar (i.e., Mace or Arapahoe), either healthy (control) or infected with virus (i.e., TriMV or WSMV, or double-infection with both viruses), at a specific temperature (i.e., 18 °C or 27 °C). First, the FastQC [40] tool was used to ensure each sequence set had a good “per base quality” score, and we found that all reads had Phred quality score > 30. Next, we employed the Cutadapt [41] tool to remove the residual Illumina TruSeq sequencing adapters using the sequence “TGGAATTCTCGGGTGCCAAGG” with an error rate of 0.1. Finally, the Cutadapt tool was used once more to trim down the reads to a range of 18–36 base pairs, which is a range reasonable for miRNA (21-25 bp) detection while also being computationally less challenging. The resulting datasets were used as query input for alignment against the mature miRNA database.

### Building a database of wheat mature miRNA transcripts, aligning and quantifying

For wheat, 125 mature miRNA sequences were available as of December 2022 in the curated miRBAse database [42]. This set of miRNAs was missing eight of the highly conserved miRNA families [25, 43]: miR166, miR168, miR170/171, miR172, miR390, miR393, miR394, and miR444. The missing sequences were added to the original set and used to build the reference miRNA database using the bowtie2 (version 2.4, [44]). We mapped our preprocessed datasets against the reference miRNA database using bowtie2 in default settings to determine the miRNA abundance between the samples. The reads that did not align with our reference miRNA database were considered different types of RNA (i.e., rRNA, tRNA, and mRNA) and discarded from the downstream analysis. It is possible that there were yet unidentified miRNAs among the discarded reads. However, in contrast to identifying new miRNAs, the purpose of this work is to determine the changes in the miRNA profiles of wheat cultivars in the presence of viral infection using existing knowledge-based approaches. And thus, we limited our study to miRBase-derived reference database of 137 miRNAs which we believe computationally enough to unravel miRNA differential expression in response to viral infections. miRNA reads of wheat infected with WSMV, TriMV, double infection, or healthy plants were quantified using *Salmon* [45] in the alignment mode and default settings.

### Differential expression and statistical analysis

A custom in-house script was developed to import, process the data, run statistical analyses, and visualize results in R (version 4.1.2,, [46]). In brief, the count tables (i.e., the output of Salmon) were imported using *tximport* [47]. Transcripts with a total row sum of less than 40, corresponding to low expression (i.e., less than 5 for a particular treatment, [48]), were filtered out to enhance reliability. Normalization of the library was carried out using DeSEQ2 [49]. Fold changes for each mature miRNA transcript were determined by comparing normalized count values between infected and control samples. Principal component analysis, confidence values such as eigenvalues (library: FactoMineR [50]), cumulative variance (library: FactoMineR), Kaiser–Meyer–Olkin (KMO) measure (library: EFAtools [51]), and Bartlett’s Test of Sphericity (BTS) (library: EFAtools) were all computed using built-in functions. For cluster analysis, the K-Means algorithm was utilized. The optimal cluster size was determined through the silhouette method. The miRNAs showing a strong correlation with symptomatic wheat cultivars were determined from the loadings observed in the first two principal components.

### Viruses

To use in the validation experiment, in vitro transcripts of WSMV isolate Sidney 81 and TriMV isolate Nebraska were inoculated onto wheat cv. Tomahawk at the single-leaf stage [52, 53]. WSMV- and TriMV-infected wheat leaves were collected at 14 dpi, and stored in 0.5 g aliquots at -80 °C.

### Inoculation of wheat cultivars with WSMV, TriMV, or WSMV + TriMV

WSMV, TriMV, and WSMV + TriMV inocula were prepared by grinding virus-infected wheat leaves in 20 mM sodium phosphate buffer, pH 7.0 at 1:20 (W/V) dilution. Wheat cvs. Arapahoe and Mace were inoculated with crude extract of WSMV, TriMV, or WSMV + TriMV in 20 mM sodium phosphate buffer, pH 7.0, at the single leaf stage. Virus-inoculated wheat seedlings were incubated at 27 °C and 18 °C with a 16 h photoperiod in growth chambers (Percival, IA, USA). Wheat cvs. Arapahoe and Mace seedlings inoculated with 20 mM sodium phosphate buffer, pH 7.0, were used as mock-inoculated controls. Wheat cv. Arapahoe inoculated with WSMV or TriMV elicited chlorotic streaks, mosaic, and mottling symptoms at 7 dpi at 27 °C and 18 °C, while wheat co-inoculated with WSMV + TriMV elicited severe chlorotic streaks and mosaic symptoms at 10–14 dpi. At 27 °C, wheat cv. Mace inoculated with WSMV or TriMV developed mild chlorotic streaks and mosaic symptoms at 7 dpi, and Mace co-inoculated with WSMV + TriMV developed moderate to severe chlorotic streaks and mosaic symptoms at 12–14 dpi. WSMV, TriMV, or WSMV + TriMV infected 100% of wheat cv. Arapahoe at 18 °C and 27 °C and cv. Mace at 27 °C. At 18 °C, Mace did not infect systemically by WSMV, TriMV, or WSMV + TriMV. Fully expanded upper leaves of wheat cvs. Arapahoe and Mace inoculated with WSMV, TriMV, WSMV + TriMV, or mock were collected for RNA isolation at 16 dpi.

### Total RNA isolation, polyadenylation, reverse transcription, and Real-Time PCR amplification of miRNAs

Total RNA was isolated from fully expanded upper leaves of wheat infected with WSMV, TriMV, WSMV plus TriMV, or mock-inoculated wheat plants using TRIpure reagent (Roche, IN, USA), followed by treatment with DNAse I (GoldBio, MO, USA). One microgram of the total RNA was polyadenylated using *E. coli* Poly(A) polymerase (New England Biolabs, MA, USA), and the first-strand cDNA was subsequently synthesized using ProtoScript II Reverse Transcriptase (New England Biolabs, MA, USA) and poly(T) adapter (Table S4).

For RT-qPCR, 1 ng template cDNA, miRNA-specific forward miRNA primer, and pTR (5 mM each) (Table S4) were mixed with PowerTrack SYBR Green Master Mix (Thermo Fisher Scientific, MA, USA) in a final volume of 20 µl. Three biological replicates were employed for each treatment, with two technical replicates for each biological replicate. BioRad CFX96 was used with a standard-short protocol for the amplification of miRNA templates. Briefly, a single step of 2 min at 95 °C was used for enzyme activation, followed by 40 cycles of 15 s at 95 °C and 30 s at 55 °C, and a standard dissociation step (i.e., 65 °C to 95 °C, 0.5 °C increments). For each biological replicate, gene expression (ΔCt) was calculated by subtracting Ct values of the normalization gene (5.8S ribosomal RNA) from Ct values of inoculated (virus- or mock-) samples.

### Potential miRNA target sites detection and function analysis

To identify potential miRNA target sites, we employed mature miRNA sequences from the reference miRNA database to query the Wheat RefSeq RNA sequences (IWGSC CS RefSeq v2.1). To ensure a comprehensive search, we utilized specific parameters within the BlastN algorithm. Firstly, the search was executed on the minus strand, considering the complementary nature of miRNA:target interactions. Additionally, to address the challenge of high e-values associated with short alignments, we adjusted the e-value threshold to 100. Moreover, to encompass partially mismatched alignments, we increased the reward value from 1 to 2. Subsequent to the BLAST analysis, we applied filters using custom Python scripts. The predicted targets underwent further refinement based on a set of established criteria, which combined rules from prior studies [34, 35]. In summary, the filtering criteria comprised the following; 1) no more than four mismatches between the miRNA and the predicted target, 2) the absence of adjacent mismatches within positions 2–12 of the miRNA/target duplex, 3) no mismatches within positions 10–11 of the miRNA/target duplex, 4) no more than 2.5 mismatches within positions 1–12 of the miRNA/target duplex, and 5) exclusion of targets with less than 16 base pairs of complementarity. The selected targets were subsequently imported into Blast2GO [54]. Within this program, we conducted searches (BlastX), performed mapping, facilitated annotation, and generated visualizations.

### Supplementary Information

**Supplementary Material 1. **

## Data Availability

The datasets analyzed during the current study are available in the Gene Expression Omnibus (GEO) repository: Arapahoe 27 °C Control, GSM1306034; Arapahoe 18 °C Control, GSM1306035; Mace 27 °C Control, GSM1306036; Mace 18 °C Control, GSM1306037; Arapahoe 27 °C WSMV, GSM1306038; Arapahoe 18 °C WSMV, GSM1306039; Mace 27 °C WSMV, GSM1306040; Mace 18 °C WSMV, GSM1306041; Arapahoe 27 °C TriMV, GSM1306042; Arapahoe 18 °C TriMV, GSM1306043; Mace 27 °C TriMV, GSM1306044; Mace 18 °C TriMV, GSM1306045; Arapahoe 27 °C WSMV + TriMV, GSM1306046; Arapahoe 18 °C WSMV + TriMV, GSM1306047; Mace 27 °C WSMV + TriMV, GSM1306048; Mace 18 °C WSMV + TriMV, GSM1306049.
