# Supplementary material for: Differential regulation of miRNAs involved in the susceptible and resistance responses of wheat cultivars to wheat streak mosaic virus and Triticum mosaic virus
Source: BMC Genomics. 2024 Feb 28;25:221. doi: 10.1186/s12864-024-10128-1 (PMC10900693; doi:10.1186/s12864-024-10128-1)
Supplement: Supplementary file 1 — Supplementary Material 1. [file 12864_2024_10128_MOESM1_ESM.pdf]

**Table S1. The normalized miRNA abundance of wheat cultivars Arapahoe and Mace infected with WSMV, TriMV, or both at different temperatures and with viral infections.** A18: Arapahoe control at 18°C; A27°C: Arapahoe control at 27°C; M18: Mace control at 18°C; M27°C: Mace control at 27°C;  T: treatments with *Triticum mosaic virus*; W: treatments with *Wheat streak mosaic virus*; WT: treatments with both *Triticum mosaic virus and Wheat streak mosaic virus*.

| tae-miR | A18 | A18_T | A18_W | A18_TW | A27 | A27_T | A27_W | A27_TW | M18 | M18_T | M18_W | M18_TW | M27 | M27_W | M27_T | M27_TW |
|---|---|---|---|---|---|---|---|---|---|---|---|---|---|---|---|---|
| 156 | 16837.24 | 14698.88 | 19500.79 | 8687.27 | 32085.72 | 8216.70 | 13852.61 | 9994.28 | 15037.87 | 16657.54 | 26059.70 | 15327.37 | 10208.42 | 27181.32 | 9899.16 | 7800.82 |
| 159a | 49569.12 | 103792.73 | 124450.37 | 167544.82 | 52553.53 | 72148.31 | 48050.41 | 58256.78 | 53197.15 | 90960.61 | 68226.83 | 100668.53 | 75266.62 | 132896.52 | 127384.10 | 227105.09 |
| 159b | 45801.21 | 93538.87 | 112176.44 | 154147.80 | 51994.42 | 65354.67 | 43446.60 | 54295.21 | 47968.95 | 82099.70 | 62570.87 | 91111.33 | 69612.55 | 121112.13 | 116811.19 | 209269.64 |
| 160 | 291.11 | 99.58 | 126.71 | 97.80 | 163.18 | 128.13 | 138.92 | 104.85 | 265.03 | 532.14 | 233.85 | 344.04 | 275.33 | 76.68 | 111.41 | 43.02 |
| 164 | 955.79 | 671.20 | 412.69 | 401.41 | 668.30 | 654.28 | 181.37 | 237.08 | 1047.68 | 1800.01 | 968.39 | 2516.84 | 1412.30 | 141.62 | 599.47 | 329.12 |
| 166 | 282703.46 | 176225.17 | 188464.44 | 56801.05 | 566592.15 | 175669.47 | 97477.63 | 44734.42 | 277617.81 | 318013.62 | 773330.34 | 363687.89 | 151668.58 | 117196.78 | 69239.33 | 33620.15 |
| 167a | 145.97 | 88.86 | 65.09 | 51.03 | 137.98 | 98.14 | 148.36 | 82.94 | 131.38 | 118.54 | 113.93 | 130.81 | 113.37 | 74.33 | 79.58 | 55.02 |
| 167c-5p | 7809.60 | 7156.27 | 8912.90 | 6278.82 | 13777.59 | 6154.80 | 8034.91 | 6412.99 | 9733.78 | 11190.49 | 15359.39 | 11389.90 | 9165.39 | 12043.38 | 6206.87 | 9043.27 |
| 168a | 37725.24 | 172829.48 | 315989.88 | 277966.27 | 24532.82 | 146750.17 | 282748.90 | 200306.47 | 47382.25 | 51418.29 | 57755.88 | 69440.83 | 32137.96 | 296755.90 | 61727.42 | 256193.60 |
| 171 | 202.87 | 80.67 | 67.86 | 44.22 | 130.78 | 252.63 | 130.35 | 115.02 | 171.03 | 302.79 | 214.37 | 272.09 | 212.17 | 65.73 | 122.02 | 70.03 |
| 171a | 215.24 | 85.08 | 72.70 | 43.37 | 149.98 | 255.35 | 138.07 | 125.19 | 177.82 | 282.18 | 227.86 | 266.86 | 242.94 | 71.98 | 122.02 | 43.02 |
| 171b | 9.90 | 28.36 | 13.16 | 15.31 | 14.40 | 56.34 | 18.44 | 33.65 | 6.80 | 9.02 | 10.49 | 2.62 | 22.67 | 14.08 | 10.61 | 21.01 |
| 172 | 78.34 | 26.47 | 22.85 | 11.06 | 77.99 | 32.71 | 23.15 | 21.13 | 58.90 | 99.21 | 76.45 | 95.49 | 116.61 | 23.47 | 42.44 | 13.00 |
| 319 | 87.41 | 40.97 | 28.39 | 16.16 | 327.55 | 145.40 | 35.59 | 24.26 | 79.28 | 110.81 | 131.92 | 73.26 | 178.16 | 35.21 | 63.66 | 25.01 |
| 390 | 37.11 | 11.97 | 24.93 | 7.65 | 81.59 | 89.96 | 86.61 | 61.03 | 37.38 | 37.37 | 53.97 | 28.78 | 38.87 | 55.55 | 21.22 | 17.01 |
| 393 | 1900.03 | 4097.13 | 1717.91 | 3673.91 | 1097.84 | 4073.82 | 4012.53 | 5684.52 | 2712.64 | 3449.27 | 2822.73 | 2624.11 | 2960.65 | 2148.59 | 2914.22 | 3776.36 |
| 394 | 235.85 | 94.53 | 97.63 | 79.94 | 161.98 | 200.83 | 78.90 | 113.46 | 300.15 | 708.67 | 373.27 | 325.72 | 413.00 | 104.85 | 344.83 | 120.04 |
| 395a | 7.42 | 3.15 | 4.85 | 7.65 | 19.20 | 36.35 | 955.75 | 84.50 | 6.80 | 2.58 | 3.00 | 26.16 | 17.82 | 14.08 | 203.36 | 16.01 |
| 395b | 13.19 | 5.67 | 9.69 | 11.91 | 10.80 | 38.17 | 844.70 | 86.07 | 13.59 | 3.87 | 3.00 | 19.62 | 17.82 | 10.17 | 104.33 | 8.00 |
| 396-5p | 9.90 | 39.07 | 13.16 | 26.36 | 9.60 | 185.38 | 76.75 | 161.97 | 10.19 | 10.31 | 8.99 | 11.77 | 14.58 | 85.29 | 60.12 | 94.03 |
| 397-5p | 26.39 | 698.30 | 282.51 | 386.10 | 53.99 | 472.54 | 546.27 | 332.54 | 6.80 | 12.88 | 35.98 | 31.40 | 24.29 | 6703.98 | 2463.30 | 2433.88 |
| 398 | 24.74 | 677.50 | 193.88 | 299.36 | 61.19 | 197.19 | 209.67 | 187.79 | 56.63 | 137.87 | 130.42 | 116.42 | 119.85 | 4902.79 | 3409.36 | 3159.14 |
| 408 | 18.14 | 31.51 | 31.85 | 65.48 | 43.19 | 13.63 | 11.58 | 8.61 | 15.86 | 33.50 | 70.46 | 62.79 | 38.87 | 616.57 | 178.60 | 556.20 |
| 444a-2 | 166.58 | 149.99 | 216.73 | 216.86 | 202.77 | 79.97 | 143.21 | 112.67 | 81.55 | 150.75 | 122.92 | 143.89 | 173.30 | 141.62 | 65.43 | 94.03 |
| 444b-1 | 257.30 | 550.19 | 539.40 | 585.95 | 393.54 | 352.59 | 356.75 | 424.09 | 225.39 | 184.25 | 329.79 | 214.53 | 181.40 | 368.53 | 291.78 | 532.19 |
| 444b-2 | 104.73 | 91.38 | 180.72 | 119.91 | 184.77 | 56.34 | 157.36 | 86.85 | 72.49 | 65.71 | 92.94 | 53.63 | 80.98 | 157.27 | 74.27 | 66.02 |
| 444c-1 | 209.47 | 530.65 | 492.31 | 541.73 | 435.54 | 297.15 | 350.31 | 412.35 | 231.06 | 163.64 | 323.80 | 210.61 | 195.97 | 384.18 | 251.10 | 489.18 |
| 444c-2 | 91.54 | 74.37 | 170.34 | 115.66 | 153.58 | 64.52 | 158.22 | 88.42 | 66.82 | 90.19 | 119.92 | 65.41 | 100.42 | 155.71 | 56.59 | 86.03 |
| 531 | 41.23 | 15.76 | 24.23 | 3.40 | 21.60 | 20.90 | 3.00 | 18.00 | 58.90 | 81.17 | 67.46 | 2.62 | 158.72 | 31.30 | 122.02 | 29.01 |
| 1117 | 15.67 | 13.23 | 20.08 | 17.01 | 7.20 | 30.90 | 30.87 | 19.56 | 9.06 | 16.75 | 14.99 | 7.85 | 14.58 | 28.95 | 10.61 | 20.01 |
| 1118 | 0.82 | 3.78 | 4.15 | 5.10 | 0.00 | 0.91 | 3.43 | 3.13 | 1.13 | 1.29 | 0.00 | 0.00 | 1.62 | 3.91 | 1.77 | 1.00 |
| 1120a | 9.07 | 2.52 | 8.31 | 4.25 | 12.00 | 5.45 | 5.15 | 7.04 | 9.06 | 7.73 | 11.99 | 13.08 | 69.64 | 8.61 | 12.38 | 10.00 |
| 1120b-3p | 14.84 | 8.82 | 10.39 | 4.25 | 7.20 | 10.00 | 6.43 | 12.52 | 14.72 | 9.02 | 16.49 | 14.39 | 14.58 | 7.82 | 5.31 | 9.00 |
| 1120c-5p | 18.97 | 18.28 | 47.78 | 54.43 | 18.00 | 16.36 | 21.01 | 26.60 | 13.59 | 11.60 | 7.50 | 10.47 | 14.58 | 18.78 | 19.45 | 19.01 |
| 1121 | 4.12 | 11.34 | 4.85 | 9.35 | 7.20 | 9.09 | 8.58 | 9.39 | 11.33 | 9.02 | 4.50 | 6.54 | 8.10 | 6.26 | 12.38 | 12.00 |
| 1122b-3p | 7.42 | 9.45 | 15.93 | 21.26 | 13.20 | 8.18 | 15.01 | 25.82 | 14.72 | 9.02 | 7.50 | 13.08 | 17.82 | 16.43 | 22.99 | 23.01 |
| 1125 | 10.72 | 20.80 | 24.23 | 10.21 | 14.40 | 10.90 | 18.87 | 9.39 | 12.46 | 7.73 | 17.99 | 17.01 | 14.58 | 14.08 | 3.54 | 7.00 |
| 1127b-3p | 50.30 | 62.39 | 51.24 | 62.08 | 33.60 | 62.70 | 50.60 | 67.29 | 49.84 | 51.54 | 53.97 | 44.48 | 64.78 | 70.42 | 90.19 | 117.04 |
| 1130a | 34.64 | 54.83 | 51.93 | 37.42 | 28.80 | 34.53 | 33.44 | 18.78 | 49.84 | 55.40 | 47.97 | 48.40 | 29.15 | 52.42 | 40.67 | 40.01 |
| 1130b-3p | 26.39 | 47.90 | 38.78 | 45.07 | 18.00 | 39.98 | 26.16 | 40.69 | 45.31 | 42.52 | 50.97 | 66.71 | 38.87 | 33.65 | 76.04 | 55.02 |
| 1133 | 8.25 | 4.41 | 6.23 | 12.76 | 9.60 | 18.17 | 2.57 | 8.61 | 7.93 | 3.87 | 7.50 | 9.16 | 1.62 | 5.48 | 17.68 | 1.00 |
| 1135 | 359.56 | 766.36 | 923.70 | 1127.69 | 325.15 | 568.86 | 727.64 | 842.70 | 432.66 | 630.07 | 704.56 | 724.70 | 678.62 | 1089.94 | 1098.14 | 1470.53 |
| 1136 | 86.59 | 80.04 | 83.09 | 78.24 | 59.99 | 57.25 | 66.03 | 64.16 | 96.27 | 127.56 | 103.44 | 142.59 | 59.93 | 68.07 | 51.28 | 48.02 |
| 1137a | 5.77 | 22.69 | 22.16 | 28.06 | 4.80 | 17.27 | 29.16 | 25.04 | 56.63 | 6.44 | 4.50 | 6.54 | 195.97 | 34.43 | 311.23 | 22.01 |
| 1137b-5p | 143.49 | 163.23 | 173.11 | 136.92 | 129.58 | 151.76 | 145.79 | 130.67 | 171.03 | 175.23 | 232.35 | 188.37 | 140.91 | 195.61 | 175.07 | 144.05 |
| 1139 | 1.65 | 2.52 | 1.38 | 0.85 | 1.20 | 3.63 | 4.72 | 3.91 | 0.00 | 0.00 | 0.00 | 1.31 | 1.62 | 7.82 | 0.00 | 8.00 |
| 1847-5p | 322.45 | 245.16 | 256.89 | 171.79 | 167.98 | 137.22 | 129.49 | 110.33 | 400.95 | 296.35 | 625.11 | 249.85 | 126.33 | 165.10 | 111.41 | 77.03 |

| | | | | | | | | | | | | | | | | |
|---|---|---|---|---|---|---|---|---|---|---|---|---|---|---|---|---|
| **5048-5p** | 12341.97 | 9145.91 | 9372.68 | 9339.56 | 10048.53 | 13712.68 | 8095.37 | 8669.58 | 14345.83 | 17367.49 | 13950.28 | 14891.76 | 12020.76 | 10144.38 | 18606.47 | 10687.86 |
| **5049-3p** | 45.36 | 53.57 | 49.85 | 30.62 | 52.79 | 49.98 | 24.87 | 37.56 | 44.17 | 38.65 | 34.48 | 32.70 | 42.11 | 19.56 | 44.21 | 31.01 |
| **5050** | 25.56 | 24.58 | 8.31 | 13.61 | 20.40 | 17.27 | 27.87 | 28.95 | 30.58 | 25.77 | 16.49 | 19.62 | 22.67 | 3.13 | 8.84 | 12.00 |
| **5062-5p** | 209.47 | 86.97 | 217.42 | 136.92 | 81.59 | 47.25 | 35.16 | 21.91 | 244.65 | 306.66 | 250.34 | 264.24 | 140.91 | 77.46 | 54.82 | 84.03 |
| **5084** | 2.47 | 5.67 | 9.00 | 9.35 | 4.80 | 10.00 | 5.57 | 6.26 | 4.53 | 6.44 | 4.50 | 2.62 | 9.72 | 4.69 | 1.77 | 6.00 |
| **5175-5p** | 776.84 | 503.55 | 390.53 | 219.41 | 664.70 | 362.58 | 138.07 | 251.17 | 911.76 | 903.23 | 884.45 | 903.92 | 1075.42 | 179.96 | 449.16 | 186.07 |
| **5200** | 2881.39 | 1583.14 | 1602.97 | 1300.33 | 2771.60 | 1054.12 | 1575.77 | 2136.10 | 3425.06 | 4516.14 | 3698.19 | 3896.92 | 5399.79 | 932.67 | 1538.46 | 933.34 |
| **5384-3p** | 10.72 | 109.66 | 362.14 | 275.54 | 24.00 | 105.41 | 240.55 | 194.05 | 12.46 | 27.06 | 31.48 | 34.01 | 16.20 | 320.02 | 33.60 | 286.10 |
| **6197-5p** | 18.97 | 25.84 | 19.39 | 47.62 | 3.60 | 17.27 | 11.58 | 18.78 | 19.25 | 23.19 | 13.49 | 20.93 | 8.10 | 11.74 | 31.83 | 55.02 |
| **7757-5p** | 9538.93 | 7473.91 | 10724.98 | 10258.04 | 25933.02 | 8452.06 | 7074.45 | 7145.36 | 15473.93 | 14843.35 | 20857.95 | 21156.40 | 13515.66 | 9223.45 | 21299.65 | 11696.23 |
| **6201** | 7.42 | 2.52 | 2.08 | 0.85 | 24.00 | 3.63 | 8.58 | 3.91 | 7.93 | 6.44 | 16.49 | 6.54 | 11.34 | 4.69 | 3.54 | 1.00 |
| **9652-3p** | 0.00 | 0.63 | 4.85 | 3.40 | 0.00 | 6.36 | 5.57 | 9.39 | 0.00 | 0.00 | 0.00 | 1.31 | 1.62 | 16.43 | 1.77 | 29.01 |
| **9652-5p** | 319.15 | 82.56 | 126.71 | 127.57 | 111.58 | 58.16 | 59.60 | 62.60 | 477.97 | 427.78 | 410.74 | 463.08 | 171.68 | 133.80 | 159.15 | 169.06 |
| **9653a-3p** | 3837.18 | 10955.93 | 10881.47 | 10625.43 | 8524.76 | 10185.00 | 14946.00 | 13730.49 | 4095.57 | 4806.05 | 5572.02 | 4974.82 | 9490.93 | 15421.19 | 9773.61 | 20963.58 |
| **9653b** | 264.72 | 97.69 | 148.18 | 39.12 | 925.07 | 279.89 | 148.36 | 180.75 | 334.12 | 402.01 | 599.62 | 242.00 | 1229.29 | 244.90 | 893.01 | 74.03 |
| **9654a-3p** | 4.12 | 2.52 | 1.38 | 0.85 | 0.00 | 19.08 | 6.00 | 2.35 | 1.13 | 5.15 | 0.00 | 10.47 | 0.00 | 3.13 | 1.77 | 1.00 |
| **9654b-3p** | 138.54 | 27.10 | 51.93 | 21.26 | 98.39 | 40.89 | 25.73 | 16.43 | 58.90 | 55.40 | 97.44 | 57.56 | 30.77 | 9.39 | 22.99 | 4.00 |
| **9655-3p** | 21.44 | 64.91 | 56.09 | 42.52 | 40.79 | 365.31 | 246.98 | 78.25 | 13.59 | 7.73 | 17.99 | 9.16 | 16.20 | 121.28 | 22.99 | 27.01 |
| **9656-3p** | 59.38 | 2865.66 | 592.72 | 838.54 | 64.79 | 2892.48 | 977.19 | 1640.02 | 122.32 | 134.00 | 131.92 | 146.51 | 158.72 | 374.01 | 825.81 | 1052.38 |
| **9657a-3p** | 9.90 | 101.47 | 118.40 | 199.00 | 3.60 | 129.04 | 114.48 | 251.95 | 16.99 | 11.60 | 17.99 | 32.70 | 37.25 | 308.28 | 270.56 | 774.28 |
| **9657b-3p** | 20.62 | 35.29 | 51.93 | 61.23 | 4.80 | 32.71 | 28.73 | 46.95 | 32.85 | 11.60 | 28.48 | 15.70 | 11.34 | 49.29 | 28.29 | 81.03 |
| **9657c-3p** | 18.97 | 35.92 | 63.70 | 68.89 | 4.80 | 33.62 | 31.73 | 51.64 | 31.71 | 29.64 | 19.49 | 27.47 | 32.39 | 50.08 | 45.98 | 93.03 |
| **9658-3p** | 178.13 | 142.43 | 516.55 | 310.41 | 182.37 | 150.85 | 237.54 | 111.89 | 127.99 | 134.00 | 244.35 | 146.51 | 72.88 | 424.87 | 129.09 | 283.10 |
| **9660-5p** | 31.34 | 30.88 | 167.57 | 134.37 | 28.80 | 31.81 | 32.16 | 29.73 | 22.65 | 24.48 | 49.47 | 37.94 | 11.34 | 97.02 | 5.31 | 94.03 |
| **9661-5p** | 342.24 | 136.13 | 303.97 | 288.30 | 208.77 | 215.37 | 442.50 | 442.87 | 133.65 | 171.37 | 136.41 | 108.57 | 272.09 | 396.70 | 314.76 | 467.17 |
| **9662a-3p** | 8942.69 | 7354.16 | 9770.82 | 8332.64 | 10395.28 | 7506.99 | 6338.66 | 5918.48 | 12255.01 | 15532.69 | 17156.77 | 15306.44 | 12221.59 | 10501.18 | 13301.45 | 10865.93 |
| **9662b-3p** | 3902.33 | 1674.52 | 3127.69 | 1338.60 | 2300.06 | 999.60 | 832.26 | 513.29 | 3559.84 | 4912.99 | 4785.01 | 4080.06 | 1436.60 | 747.23 | 1257.29 | 613.22 |
| **9663-5p** | 2205.99 | 1650.57 | 1105.80 | 3217.22 | 955.06 | 1637.52 | 1935.51 | 3539.04 | 974.06 | 798.86 | 709.06 | 732.55 | 118.23 | 74.33 | 114.94 | 160.06 |
| **9664-3p** | 84.94 | 186.55 | 16.62 | 200.70 | 86.39 | 149.03 | 31.30 | 109.54 | 9.06 | 15.46 | 11.99 | 11.77 | 16.20 | 64.94 | 111.41 | 199.07 |
| **9666a-3p** | 204.52 | 118.48 | 169.64 | 229.62 | 629.91 | 258.99 | 313.01 | 575.10 | 15.86 | 23.19 | 29.98 | 26.16 | 77.74 | 111.11 | 51.28 | 76.03 |
| **9666b-3p** | 94.01 | 41.60 | 22.85 | 23.81 | 105.58 | 210.82 | 251.27 | 383.40 | 19.25 | 68.29 | 26.98 | 74.56 | 197.59 | 194.83 | 111.41 | 455.16 |
| **9666b-5p** | 17.32 | 22.69 | 2.77 | 2.55 | 18.00 | 39.98 | 69.89 | 69.64 | 6.80 | 5.15 | 8.99 | 2.62 | 19.44 | 61.03 | 17.68 | 44.02 |
| **9666c-5p** | 19.79 | 24.58 | 6.92 | 6.80 | 20.40 | 30.90 | 70.75 | 68.07 | 2.27 | 1.29 | 6.00 | 2.62 | 16.20 | 61.03 | 30.06 | 55.02 |
| **9668-5p** | 9.07 | 9.45 | 6.92 | 19.56 | 10.80 | 7.27 | 11.58 | 19.56 | 12.46 | 11.60 | 3.00 | 9.16 | 11.34 | 11.74 | 15.92 | 13.00 |
| **9669-5p** | 0.00 | 1.89 | 0.00 | 0.00 | 9.60 | 39.08 | 50.60 | 54.77 | 278.63 | 296.35 | 670.08 | 310.03 | 168.44 | 1710.42 | 307.69 | 754.27 |
| **9670-3p** | 44.53 | 1304.58 | 1432.63 | 1234.84 | 75.59 | 1005.05 | 1517.02 | 1963.96 | 23.79 | 55.40 | 62.96 | 79.80 | 93.94 | 2250.31 | 1457.11 | 2153.78 |
| **9672a-3p** | 3488.34 | 5482.38 | 3795.19 | 3734.29 | 2237.67 | 3313.22 | 5534.70 | 6438.81 | 1420.31 | 1637.66 | 2446.47 | 1619.47 | 981.48 | 1148.63 | 664.90 | 957.35 |
| **9672b** | 10680.27 | 20623.68 | 13629.71 | 11721.65 | 30665.13 | 16049.01 | 13602.20 | 12081.86 | 9165.20 | 8255.32 | 20544.65 | 13268.37 | 9388.89 | 5704.02 | 4686.10 | 5339.93 |
| **9673-5p** | 58.55 | 25.21 | 13.85 | 25.51 | 56.39 | 25.44 | 49.31 | 49.29 | 71.36 | 61.85 | 44.97 | 68.02 | 74.50 | 76.68 | 83.11 | 88.03 |
| **9674b-5p** | 8728.28 | 7063.63 | 11103.74 | 9528.36 | 11601.11 | 9584.33 | 6845.91 | 6762.74 | 11707.95 | 16354.74 | 16206.36 | 17959.33 | 14662.35 | 10516.04 | 13373.95 | 10170.68 |
| **9675-3p** | 113.80 | 73.11 | 53.32 | 43.37 | 116.38 | 261.71 | 153.93 | 102.50 | 28.32 | 34.79 | 28.48 | 53.63 | 32.39 | 27.39 | 30.06 | 11.00 |
| **9676-5p** | 56.08 | 470.15 | 96.94 | 155.63 | 79.19 | 189.02 | 116.63 | 222.22 | 58.90 | 148.18 | 98.94 | 156.98 | 221.89 | 323.15 | 687.88 | 999.36 |
| **9677a** | 10.72 | 5.04 | 4.85 | 0.85 | 13.20 | 7.27 | 16.29 | 13.30 | 9.06 | 10.31 | 7.50 | 11.77 | 9.72 | 9.39 | 0.00 | 3.00 |
| **9679-5p** | 447.79 | 189.07 | 281.82 | 181.99 | 1118.24 | 199.92 | 154.79 | 89.20 | 550.46 | 566.93 | 881.45 | 541.57 | 630.03 | 274.64 | 265.25 | 211.08 |
| **9772** | 1294.73 | 724.14 | 1039.33 | 1349.65 | 1724.15 | 1104.10 | 1577.05 | 1371.64 | 1167.74 | 1861.86 | 1521.55 | 2079.93 | 1319.98 | 1081.34 | 1099.91 | 1725.62 |
| **9773** | 1514.09 | 211.76 | 301.21 | 267.04 | 1817.73 | 317.15 | 177.52 | 163.53 | 1073.73 | 1185.41 | 941.41 | 1378.77 | 1243.86 | 133.02 | 866.49 | 58.02 |
| **9774** | 4089.53 | 1246.60 | 2630.53 | 858.95 | 12754.14 | 4061.10 | 2007.98 | 2080.54 | 3610.81 | 2971.25 | 4554.15 | 2957.68 | 10415.73 | 2429.49 | 8312.96 | 517.19 |
| **9775** | 140.19 | 160.08 | 369.76 | 398.86 | 28.80 | 56.34 | 174.51 | 154.14 | 37.38 | 34.79 | 28.48 | 35.32 | 12.96 | 90.76 | 37.14 | 102.04 |
| **9776** | 309.25 | 240.75 | 297.05 | 205.81 | 793.08 | 508.89 | 179.66 | 241.00 | 266.17 | 181.68 | 377.76 | 253.78 | 262.38 | 266.81 | 291.78 | 303.11 |
| **9778** | 7.42 | 6.30 | 2.77 | 0.00 | 1.20 | 2.73 | 8.15 | 6.26 | 11.33 | 3.87 | 20.99 | 11.77 | 4.86 | 6.26 | 1.77 | 0.00 |
| **9779** | 14.84 | 5.67 | 8.31 | 0.00 | 19.20 | 9.09 | 2.57 | 3.13 | 7.93 | 6.44 | 14.99 | 11.77 | 16.20 | 5.48 | 12.38 | 1.00 |
| **9781** | 0.82 | 3.78 | 4.85 | 11.06 | 1.20 | 11.81 | 12.01 | 14.87 | 6.80 | 11.60 | 6.00 | 10.47 | 8.10 | 48.51 | 111.41 | 89.03 |
| **9782** | 23.09 | 13.23 | 31.16 | 33.17 | 37.19 | 26.35 | 52.74 | 53.21 | 21.52 | 27.06 | 22.49 | 27.47 | 12.96 | 50.86 | 26.53 | 35.01 |
| **9783** | 11.55 | 23.95 | 14.54 | 45.07 | 6.00 | 20.90 | 23.15 | 38.34 | 5.66 | 1.29 | 0.00 | 1.31 | 1.62 | 9.39 | 5.31 | 6.00 |

**Table S2. The differential abundance of miRNAs in wheat cultivars (cv. Arapahoe and cv. Mace) at different temperatures with double infection.** The values represent the binary logarithm of the normalized abundance counts. A18: Arapahoe control at 18°C; A27: Arapahoe control at 27°C; M18: Mace control at 18°C; M27°C: Mace control at 27°C: TW: treatments with both Triticum mosaic virus and Wheat streak mosaic virus. Diff: logarithmic subtraction to compare the abundance of a specific miRNA between the double-infected sample and the control sample.

| tae-miR | A18 | A27 | M18 | M27 | A18_TW | M18_TW | A27_TW | M27_TW | Double Infection | | | |
|---|---|---|---|---|---|---|---|---|---|---|---|---|
| | | | | | | | | | Diff(A18) | Diff(M18) | Diff(A27) | Diff(M27) |
| **156** | 14.04 | 14.97 | 13.88 | 13.32 | 13.08 | 13.90 | 13.29 | 12.93 | -0.95 | 0.03 | -1.68 | -0.39 |
| **159a** | 15.60 | 15.68 | 15.70 | 16.20 | 17.35 | 16.62 | 15.83 | 17.79 | 1.76 | 0.92 | 0.15 | 1.59 |
| **159b** | 15.48 | 15.67 | 15.55 | 16.09 | 17.23 | 16.48 | 15.73 | 17.68 | 1.75 | 0.93 | 0.06 | 1.59 |
| **160** | 8.19 | 7.35 | 8.05 | 8.11 | 6.61 | 8.43 | 6.71 | 5.43 | -1.57 | 0.38 | -0.64 | -2.68 |
| **164** | 9.90 | 9.38 | 10.03 | 10.46 | 8.65 | 11.30 | 7.89 | 8.36 | -1.25 | 1.26 | -1.50 | -2.10 |
| **166** | 18.11 | 19.11 | 18.08 | 17.21 | 15.79 | 18.47 | 15.45 | 15.04 | -2.32 | 0.39 | -3.66 | -2.17 |
| **167a** | 7.19 | 7.11 | 7.04 | 6.82 | 5.67 | 7.03 | 6.37 | 5.78 | -1.52 | -0.01 | -0.73 | -1.04 |
| **167c-5p** | 12.93 | 13.75 | 13.25 | 13.16 | 12.62 | 13.48 | 12.65 | 13.14 | -0.31 | 0.23 | -1.10 | -0.02 |
| **168a** | 15.20 | 14.58 | 15.53 | 14.97 | 18.08 | 16.08 | 17.61 | 17.97 | 2.88 | 0.55 | 3.03 | 2.99 |
| **171a** | 7.66 | 7.03 | 7.42 | 7.73 | 5.47 | 8.09 | 6.85 | 6.13 | -2.20 | 0.67 | -0.19 | -1.60 |
| **171b** | 7.75 | 7.23 | 7.47 | 7.92 | 5.44 | 8.06 | 6.97 | 5.43 | -2.31 | 0.59 | -0.26 | -2.50 |
| **171** | 3.31 | 3.85 | 2.76 | 4.50 | 3.94 | 1.39 | 5.07 | 4.39 | 0.63 | -1.38 | 1.22 | -0.11 |
| **172** | 6.29 | 6.29 | 5.88 | 6.87 | 3.47 | 6.58 | 4.40 | 3.70 | -2.83 | 0.70 | -1.88 | -3.16 |
| **319** | 6.45 | 8.36 | 6.31 | 7.48 | 4.01 | 6.19 | 4.60 | 4.64 | -2.44 | -0.11 | -3.76 | -2.83 |
| **390** | 5.21 | 6.35 | 5.22 | 5.28 | 2.94 | 4.85 | 5.93 | 4.09 | -2.28 | -0.38 | -0.42 | -1.19 |
| **393** | 10.89 | 10.10 | 11.41 | 11.53 | 11.84 | 11.36 | 12.47 | 11.88 | 0.95 | -0.05 | 2.37 | 0.35 |
| **394** | 7.88 | 7.34 | 8.23 | 8.69 | 6.32 | 8.35 | 6.83 | 6.91 | -1.56 | 0.12 | -0.51 | -1.78 |
| **395a** | 2.89 | 4.26 | 2.76 | 4.16 | 2.94 | 4.71 | 6.40 | 4.00 | 0.04 | 1.94 | 2.14 | -0.15 |
| **395b** | 3.72 | 3.43 | 3.76 | 4.16 | 3.57 | 4.29 | 6.43 | 3.00 | -0.15 | 0.53 | 2.99 | -1.15 |
| **396-5p** | 3.31 | 3.26 | 3.35 | 3.87 | 4.72 | 3.56 | 7.34 | 6.56 | 1.41 | 0.21 | 4.08 | 2.69 |
| **397-5p** | 4.72 | 5.75 | 2.76 | 4.60 | 8.59 | 4.97 | 8.38 | 11.25 | 3.87 | 2.21 | 2.62 | 6.65 |
| **398** | 4.63 | 5.94 | 5.82 | 6.91 | 8.23 | 6.86 | 7.55 | 11.63 | 3.60 | 1.04 | 1.62 | 4.72 |
| **408** | 4.18 | 5.43 | 3.99 | 5.28 | 6.03 | 5.97 | 3.11 | 9.12 | 1.85 | 1.99 | -2.33 | 3.84 |
| **444a-2** | 7.38 | 7.66 | 6.35 | 7.44 | 7.76 | 7.17 | 6.82 | 6.56 | 0.38 | 0.82 | -0.85 | -0.88 |
| **444b-1** | 8.01 | 8.62 | 7.82 | 7.50 | 9.19 | 7.75 | 8.73 | 9.06 | 1.19 | -0.07 | 0.11 | 1.55 |
| **444b-2** | 6.71 | 7.53 | 6.18 | 6.34 | 6.91 | 5.75 | 6.44 | 6.04 | 0.20 | -0.43 | -1.09 | -0.29 |
| **444c-1** | 7.71 | 8.77 | 7.85 | 7.61 | 9.08 | 7.72 | 8.69 | 8.93 | 1.37 | -0.13 | -0.08 | 1.32 |
| **444c-2** | 6.52 | 7.26 | 6.06 | 6.65 | 6.85 | 6.03 | 6.47 | 6.43 | 0.34 | -0.03 | -0.80 | -0.22 |
| **531** | 5.37 | 4.43 | 5.88 | 7.31 | 1.77 | 1.39 | 4.17 | 4.86 | -3.60 | -4.49 | -0.26 | -2.45 |
| **1117** | 3.97 | 2.85 | 3.18 | 3.87 | 4.09 | 2.97 | 4.29 | 4.32 | 0.12 | -0.21 | 1.44 | 0.46 |
| **1118** | -0.28 | 0.00 | 0.18 | 0.70 | 2.35 | 0.00 | 1.65 | 0.00 | 2.63 | -0.18 | 1.65 | -0.70 |
| **1120a** | 3.18 | 3.58 | 3.18 | 6.12 | 2.09 | 3.71 | 2.82 | 3.32 | -1.09 | 0.53 | -0.77 | -2.80 |
| **1120b-3p** | 3.89 | 2.85 | 3.88 | 3.87 | 2.09 | 3.85 | 3.65 | 3.17 | -1.80 | -0.03 | 0.80 | -0.70 |
| **1120c-5p** | 4.25 | 4.17 | 3.76 | 3.87 | 5.77 | 3.39 | 4.73 | 4.25 | 1.52 | -0.38 | 0.56 | 0.38 |
| **1121** | 2.04 | 2.85 | 3.50 | 3.02 | 3.23 | 2.71 | 3.23 | 3.59 | 1.18 | -0.79 | 0.38 | 0.57 |
| **1125** | 2.89 | 3.72 | 3.88 | 4.16 | 4.41 | 3.71 | 4.69 | 4.52 | 1.52 | -0.17 | 0.97 | 0.37 |
| **1122b-3p** | 3.42 | 3.85 | 3.64 | 3.87 | 3.35 | 4.09 | 3.23 | 2.81 | -0.07 | 0.45 | -0.62 | -1.06 |
| **1127b-3p** | 5.65 | 5.07 | 5.64 | 6.02 | 5.96 | 5.47 | 6.07 | 6.87 | 0.30 | -0.16 | 1.00 | 0.85 |
| **1130a** | 5.11 | 4.85 | 5.64 | 4.87 | 5.23 | 5.60 | 4.23 | 5.32 | 0.11 | -0.04 | -0.62 | 0.46 |

| | | | | | | | | | | | | | |
|---|---|---|---|---|---|---|---|---|---|---|---|---|---|
| **1130b-3p** | 4.72 | 4.17 | 5.50 | 5.28 | 5.49 | 6.06 | 5.35 | 5.78 | | 0.77 | 0.56 | 1.18 | 0.50 |
| **1137a** | 3.04 | 3.26 | 2.99 | 0.70 | 3.67 | 3.19 | 3.11 | 0.00 | | 0.63 | 0.21 | -0.16 | -0.70 |
| **1137b-5p** | 8.49 | 8.34 | 8.76 | 9.41 | 10.14 | 9.50 | 9.72 | 10.52 | | 1.65 | 0.74 | 1.37 | 1.12 |
| **1133** | 6.44 | 5.91 | 6.59 | 5.91 | 6.29 | 7.16 | 6.00 | 5.59 | | -0.15 | 0.57 | 0.10 | -0.32 |
| **1135** | 2.53 | 2.26 | 5.82 | 7.61 | 4.81 | 2.71 | 4.65 | 4.46 | | 2.28 | -3.11 | 2.38 | -3.15 |
| **1136** | 7.16 | 7.02 | 7.42 | 7.14 | 7.10 | 7.56 | 7.03 | 7.17 | | -0.07 | 0.14 | 0.01 | 0.03 |
| **1139** | 0.72 | 0.26 | 0.00 | 0.70 | -0.23 | 0.39 | 1.97 | 3.00 | | -0.96 | 0.39 | 1.71 | 2.30 |
| **1847-5p** | 8.33 | 7.39 | 8.65 | 6.98 | 7.42 | 7.96 | 6.79 | 6.27 | | -0.91 | -0.68 | -0.61 | -0.71 |
| **5048-5p** | 13.59 | 13.29 | 13.81 | 13.55 | 13.19 | 13.86 | 13.08 | 13.38 | | -0.40 | 0.05 | -0.21 | -0.17 |
| **5049-3p** | 5.50 | 5.72 | 5.47 | 5.40 | 4.94 | 5.03 | 5.23 | 4.95 | | -0.57 | -0.43 | -0.49 | -0.44 |
| **5050** | 4.68 | 4.35 | 4.93 | 4.50 | 3.77 | 4.29 | 4.86 | 3.59 | | -0.91 | -0.64 | 0.51 | -0.92 |
| **5062-5p** | 7.71 | 6.35 | 7.93 | 7.14 | 7.10 | 8.05 | 4.45 | 6.39 | | -0.61 | 0.11 | -1.90 | -0.75 |
| **5084** | 1.31 | 2.26 | 2.18 | 3.28 | 3.23 | 1.39 | 2.65 | 2.59 | | 1.92 | -0.79 | 0.38 | -0.70 |
| **5175-5p** | 9.60 | 9.38 | 9.83 | 10.07 | 7.78 | 9.82 | 7.97 | 7.54 | | -1.82 | -0.01 | -1.40 | -2.53 |
| **5200** | 11.49 | 11.44 | 11.74 | 12.40 | 10.34 | 11.93 | 11.06 | 9.87 | | -1.15 | 0.19 | -0.38 | -2.53 |
| **5384-3p** | 3.42 | 4.58 | 3.64 | 4.02 | 8.11 | 5.09 | 7.60 | 8.16 | | 4.68 | 1.45 | 3.02 | 4.14 |
| **6197-5p** | 4.25 | 1.85 | 4.27 | 3.02 | 5.57 | 4.39 | 4.23 | 5.78 | | 1.33 | 0.12 | 2.38 | 2.76 |
| **6201** | 13.22 | 14.66 | 13.92 | 13.72 | 13.32 | 14.37 | 12.80 | 13.51 | | 0.10 | 0.45 | -1.86 | -0.21 |
| **7757-5p** | 2.89 | 4.58 | 2.99 | 3.50 | -0.23 | 2.71 | 1.97 | 0.00 | | -3.13 | -0.28 | -2.62 | -3.50 |
| **9652-3p** | 0.00 | 0.00 | 0.00 | 0.70 | 1.77 | 0.39 | 3.23 | 4.86 | | 1.77 | 0.39 | 3.23 | 4.16 |
| **9652-5p** | 8.32 | 6.80 | 8.90 | 7.42 | 7.00 | 8.86 | 5.97 | 7.40 | | -1.32 | -0.05 | -0.83 | -0.02 |
| **9653a-3p** | 11.91 | 13.06 | 12.00 | 13.21 | 13.38 | 12.28 | 13.75 | 14.36 | | 1.47 | 0.28 | 0.69 | 1.14 |
| **9653b** | 8.05 | 9.85 | 8.38 | 10.26 | 5.29 | 7.92 | 7.50 | 6.21 | | -2.76 | -0.47 | -2.36 | -4.05 |
| **9654a-3p** | 2.04 | 0.00 | 0.18 | 0.00 | -0.23 | 3.39 | 1.23 | 0.00 | | -2.28 | 3.21 | 1.23 | 0.00 |
| **9654b-3p** | 7.11 | 6.62 | 5.88 | 4.94 | 4.41 | 5.85 | 4.04 | 2.00 | | -2.70 | -0.03 | -2.58 | -2.94 |
| **9655-3p** | 4.42 | 5.35 | 3.76 | 4.02 | 5.41 | 3.19 | 6.29 | 4.76 | | 0.99 | -0.57 | 0.94 | 0.74 |
| **9656-3p** | 5.89 | 6.02 | 6.93 | 7.31 | 9.71 | 7.19 | 10.68 | 10.04 | | 3.82 | 0.26 | 4.66 | 2.73 |
| **9657a-3p** | 3.31 | 1.85 | 4.09 | 5.22 | 7.64 | 5.03 | 7.98 | 9.60 | | 4.33 | 0.94 | 6.13 | 4.38 |
| **9657b-3p** | 4.37 | 2.26 | 5.04 | 3.50 | 5.94 | 3.97 | 5.55 | 6.34 | | 1.57 | -1.07 | 3.29 | 2.84 |
| **9657c-3p** | 4.25 | 2.26 | 4.99 | 5.02 | 6.11 | 4.78 | 5.69 | 6.54 | | 1.86 | -0.21 | 3.43 | 1.52 |
| **9658-3p** | 7.48 | 7.51 | 7.00 | 6.19 | 8.28 | 7.19 | 6.81 | 8.15 | | 0.80 | 0.20 | -0.70 | 1.96 |
| **9660-5p** | 4.97 | 4.85 | 4.50 | 3.50 | 7.07 | 5.25 | 4.89 | 6.56 | | 2.10 | 0.74 | 0.05 | 3.05 |
| **9661-5p** | 8.42 | 7.71 | 7.06 | 8.09 | 8.17 | 6.76 | 8.79 | 8.87 | | -0.25 | -0.30 | 1.08 | 0.78 |
| **9662a-3p** | 13.13 | 13.34 | 13.58 | 13.58 | 13.02 | 13.90 | 12.53 | 13.41 | | -0.10 | 0.32 | -0.81 | -0.17 |
| **9662b-3p** | 11.93 | 11.17 | 11.80 | 10.49 | 10.39 | 11.99 | 9.00 | 9.26 | | -1.54 | 0.20 | -2.16 | -1.23 |
| **9663-5p** | 11.11 | 9.90 | 9.93 | 6.89 | 11.65 | 9.52 | 11.79 | 7.32 | | 0.54 | -0.41 | 1.89 | 0.44 |
| **9664-3p** | 6.41 | 6.43 | 3.18 | 4.02 | 7.65 | 3.56 | 6.78 | 7.64 | | 1.24 | 0.38 | 0.34 | 3.62 |
| **9666a-3p** | 7.68 | 9.30 | 3.99 | 6.28 | 7.84 | 4.71 | 9.17 | 6.25 | | 0.17 | 0.72 | -0.13 | -0.03 |
| **9666b-3p** | 6.55 | 6.72 | 4.27 | 7.63 | 4.57 | 6.22 | 8.58 | 8.83 | | -1.98 | 1.95 | 1.86 | 1.20 |
| **9666b-5p** | 4.11 | 4.17 | 2.76 | 4.28 | 1.35 | 1.39 | 6.12 | 5.46 | | -2.76 | -1.38 | 1.95 | 1.18 |
| **9666c-5p** | 4.31 | 4.35 | 1.18 | 4.02 | 2.77 | 1.39 | 6.09 | 5.78 | | -1.54 | 0.21 | 1.74 | 1.76 |
| **9668-5p** | 3.18 | 3.43 | 3.64 | 3.50 | 4.29 | 3.19 | 4.29 | 3.70 | | 1.11 | -0.44 | 0.86 | 0.20 |
| **9669-5p** | 0.00 | 3.26 | 8.12 | 7.40 | 0.00 | 8.28 | 5.78 | 9.56 | | 0.00 | 0.15 | 2.51 | 2.16 |
| **9670-3p** | 5.48 | 6.24 | 4.57 | 6.55 | 10.27 | 6.32 | 10.94 | 11.07 | | 4.79 | 1.75 | 4.70 | 4.52 |
| **9672a-3p** | 11.77 | 11.13 | 10.47 | 9.94 | 11.87 | 10.66 | 12.65 | 9.90 | | 0.10 | 0.19 | 1.52 | -0.04 |
| **9672b** | 13.38 | 14.90 | 13.16 | 13.20 | 13.52 | 13.70 | 13.56 | 12.38 | | 0.13 | 0.53 | -1.34 | -0.81 |

| | | | | | | | | | | | | | |
|---|---|---|---|---|---|---|---|---|---|---|---|---|---|
| **9673-5p** | 5.87 | 5.82 | 6.16 | 6.22 | 4.67 | 6.09 | 5.62 | 6.46 | | -1.20 | -0.07 | -0.19 | 0.24 |
| **9674b-5p** | 13.09 | 13.50 | 13.52 | 13.84 | 13.22 | 14.13 | 12.72 | 13.31 | | 0.13 | 0.62 | -0.78 | -0.53 |
| **9675-3p** | 6.83 | 6.86 | 4.82 | 5.02 | 5.44 | 5.75 | 6.68 | 3.46 | | -1.39 | 0.92 | -0.18 | -1.56 |
| **9676-5p** | 5.81 | 6.31 | 5.88 | 7.79 | 7.28 | 7.29 | 7.80 | 9.96 | | 1.47 | 1.41 | 1.49 | 2.17 |
| **9677a** | 3.42 | 3.72 | 3.18 | 3.28 | -0.23 | 3.56 | 3.73 | 1.59 | | -3.66 | 0.38 | 0.01 | -1.70 |
| **9679-5p** | 8.81 | 10.13 | 9.10 | 9.30 | 7.51 | 9.08 | 6.48 | 7.72 | | -1.30 | -0.02 | -3.65 | -1.58 |
| **9772** | 10.34 | 10.75 | 10.19 | 10.37 | 10.40 | 11.02 | 10.42 | 10.75 | | 0.06 | 0.83 | -0.33 | 0.39 |
| **9773** | 10.56 | 10.83 | 10.07 | 10.28 | 8.06 | 10.43 | 7.35 | 5.86 | | -2.50 | 0.36 | -3.47 | -4.42 |
| **9774** | 12.00 | 13.64 | 11.82 | 13.35 | 9.75 | 11.53 | 11.02 | 9.01 | | -2.25 | -0.29 | -2.62 | -4.33 |
| **9775** | 7.13 | 4.85 | 5.22 | 3.70 | 8.64 | 5.14 | 7.27 | 6.67 | | 1.51 | -0.08 | 2.42 | 2.98 |
| **9776** | 8.27 | 9.63 | 8.06 | 8.04 | 7.69 | 7.99 | 7.91 | 8.24 | | -0.59 | -0.07 | -1.72 | 0.21 |
| **9778** | 2.89 | 0.26 | 3.50 | 2.28 | 0.00 | 3.56 | 2.65 | 0.00 | | -2.89 | 0.06 | 2.38 | -2.28 |
| **9779** | 3.89 | 4.26 | 2.99 | 4.02 | 0.00 | 3.56 | 1.65 | 0.00 | | -3.89 | 0.57 | -2.62 | -4.02 |
| **9781** | -0.28 | 0.26 | 2.76 | 3.02 | 3.47 | 3.39 | 3.89 | 6.48 | | 3.74 | 0.62 | 3.63 | 3.46 |
| **9782** | 4.53 | 5.22 | 4.43 | 3.70 | 5.05 | 4.78 | 5.73 | 5.13 | | 0.52 | 0.35 | 0.52 | 1.43 |
| **9783** | 3.53 | 2.58 | 2.50 | 0.70 | 5.49 | 0.39 | 5.26 | 2.59 | | 1.96 | -2.11 | 2.68 | 1.89 |

**Table S3. The calculated log2FoldChanges in miRNAs calculated for wheat (cv. Arapahoe and Mace) at different temperatures and with viral infections.**
A18: Arapahoe control at 18°C; A27°C: Arapahoe control at 27°C;  M18: Mace control at 18°C; M27°C: Mace control at 27°C; T: treatments with Triticum mosaic virus; W: treatments with Wheat streak mosaic virus; WT: treatments with both Triticum mosaic virus and Wheat streak mosaic virus

**TriMV + WSMV**

| | baseMean | log2FoldChange | pvalue |
|---|---|---|---|
| **9670-3p** | 922.21 | 4.08 | 0.000 |
| **397-5p** | 906.95 | 3.95 | 0.000 |
| **9657a-3p** | 149.84 | 3.75 | 0.000 |
| **9652-3p** | 5.02 | 3.70 | 0.001 |
| **5384-3p** | 130.19 | 3.34 | 0.000 |
| **398** | 867.74 | 2.90 | 0.002 |
| **9656-3p** | 804.78 | 2.75 | 0.000 |
| **168a** | 145728.84 | 2.28 | 0.000 |
| **396-5p** | 51.09 | 2.11 | 0.003 |
| **9781** | 22.02 | 1.92 | 0.009 |
| **9676-5p** | 255.07 | 1.43 | 0.010 |
| **408** | 112.30 | 1.36 | 0.026 |
| **9660-5p** | 51.81 | 1.28 | 0.011 |
| **9657b-3p** | 33.80 | 1.26 | 0.008 |
| **6197-5p** | 21.66 | 1.25 | 0.004 |
| **9657c-3p** | 39.94 | 1.23 | 0.005 |
| **1135** | 779.43 | 1.12 | 0.000 |
| **159a** | 97004.47 | 1.10 | 0.003 |
| **159b** | 88831.97 | 1.08 | 0.004 |
| **393** | 3104.14 | 0.76 | 0.019 |
| **1130b-3p** | 43.25 | 0.60 | 0.049 |
| **167a** | 102.21 | -0.64 | 0.035 |
| **156** | 15752.85 | -0.70 | 0.036 |
| **1120a** | 12.27 | -1.07 | 0.032 |
| **9679-5p** | 411.51 | -1.07 | 0.023 |
| **9654b-3p** | 47.30 | -1.24 | 0.020 |
| **9779** | 8.69 | -1.48 | 0.009 |
| **6201** | 6.94 | -1.62 | 0.005 |
| **531** | 43.71 | -1.65 | 0.011 |
| **9653b** | 381.42 | -1.92 | 0.002 |
| **319** | 87.69 | -1.94 | 0.001 |
| **9774** | 4093.66 | -1.94 | 0.000 |

**WSMV**

| | baseMean | log2FoldChange | pvalue |
|---|---|---|---|
| **397-5p** | 906.95 | 5.44 | 0.000 |
| **9670-3p** | 922.21 | 4.01 | 0.000 |
| **5384-3p** | 130.19 | 3.63 | 0.000 |
| **398** | 867.74 | 3.55 | 0.000 |
| **9652-3p** | 5.02 | 2.53 | 0.005 |
| **168a** | 145728.84 | 2.53 | 0.000 |
| **9657a-3p** | 149.84 | 2.33 | 0.002 |
| **9656-3p** | 804.78 | 1.72 | 0.005 |
| **9660-5p** | 51.81 | 1.46 | 0.004 |
| **9655-3p** | 72.01 | 1.23 | 0.020 |
| **9658-3p** | 212.06 | 1.16 | 0.001 |
| **396-5p** | 51.09 | 1.14 | 0.025 |
| **408** | 112.30 | 1.08 | 0.022 |
| **1117** | 17.33 | 0.83 | 0.020 |
| **1135** | 779.43 | 0.82 | 0.004 |
| **9657b-3p** | 33.80 | 0.81 | 0.043 |
| **9774** | 4093.66 | -0.94 | 0.029 |
| **1120a** | 12.27 | -0.99 | 0.027 |
| **9773** | 728.17 | -1.07 | 0.027 |
| **319** | 87.69 | -1.07 | 0.019 |

**TriMV**

| | baseMean | log2FoldChange | pvalue |
|---|---|---|---|
| **9656-3p** | 804.78 | 2.75 | 0.00 |
| **9670-3p** | 922.21 | 1.93 | 0.00 |
| **396-5p** | 51.09 | 1.67 | 0.00 |
| **9781** | 22.02 | 1.56 | 0.01 |
| **398** | 867.74 | 1.50 | 0.00 |
| **9655-3p** | 72.01 | 1.47 | 0.02 |
| **9657a-3p** | 149.84 | 1.43 | 0.00 |
| **9676-5p** | 255.07 | 1.39 | 0.01 |
| **397-5p** | 906.95 | 1.32 | 0.00 |
| **5384-3p** | 130.19 | 1.27 | 0.00 |
| **168a** | 145728.84 | 1.23 | 0.00 |
| **1135** | 779.43 | 0.71 | 0.02 |
| **393** | 3104.14 | 0.69 | 0.04 |
| **6201** | 6.94 | -1.27 | 0.02 |
| **1120a** | 12.27 | -1.42 | 0.01 |

**Table S4. Primers used in the study**

| **Name** | **Sequence** |
|---|---|
| Poly(T) adapter | GCGAGCACAGAATTAATACGACTCACTATAGG(T)$_{12}$VN |
| pTR | GCGAGCACAGAATTAATACGAC |
| Tae58-F | TGAAGAACGTAGCGAAATGC |
| Tae397F | ACCGGCGCTGCACACAAT |
| Tae398F | TGTGTTCTCAGGTCGCCC |
| Tae9670F | AGGTGGAATACTTGAAGAAGA |
| Tae9773F | TTTGTTTTTATGTTATTTTGTGAA |
| Tae5384F | CGCGCCGCCGTCGAAT |

**Table S5. Potential gene targets for miRNAs identified in this study**

<table>
<tr><th>miRNA</th><th>Target</th><th>Description</th><th>Sequence Alignment</th><th>Match Start</th><th>Match End</th></tr>
<tr><td>miR319</td><td>XM_044586284.1</td><td>transcription factor GAMYB-like(LOC123168412), transcript variant X2, mRNA</td><td>x|||||x|||||||||||||</td><td>1</td><td>19</td></tr>
<tr><td>miR319</td><td>XM_044586283.1</td><td>transcription factor GAMYB-like(LOC123168412), transcript variant X1, mRNA</td><td>x|||||x|||||||||||||</td><td>1</td><td>19</td></tr>
<tr><td>miR319</td><td>XM_044577648.1</td><td>transcription factor GAMYB-like(LOC123159833), mRNA</td><td>x|||||x|||||||||||||</td><td>1</td><td>19</td></tr>
<tr><td>miR319</td><td>XM_044567549.1</td><td>transcription factor MYB51-like(LOC123148183), transcript variant X2, mRNA</td><td>x|||||x|||||||||||||</td><td>1</td><td>19</td></tr>
<tr><td>miR319</td><td>XM_044567547.1</td><td>transcription factor MYB51-like(LOC123148183), transcript variant X1, mRNA</td><td>x|||||x|||||||||||||</td><td>1</td><td>19</td></tr>
<tr><td>miR319</td><td>XM_044502284.1</td><td>transcription factor GAMYB-like(LOC123079511), transcript variant X2, mRNA</td><td>x|||||x|||||||||||||</td><td>1</td><td>19</td></tr>
<tr><td>miR319</td><td>XM_044502283.1</td><td>transcription factor GAMYB-like(LOC123079511), transcript variant X1, mRNA</td><td>x|||||x|||||||||||||</td><td>1</td><td>19</td></tr>
<tr><td>miR319</td><td>XM_044494648.1</td><td>transcription factor GAMYB-like(LOC123071152), transcript variant X2, mRNA</td><td>x|||||x|||||||||||||</td><td>1</td><td>19</td></tr>
<tr><td>miR319</td><td>XM_044494647.1</td><td>transcription factor GAMYB-like(LOC123071152), transcript variant X1, mRNA</td><td>x|||||x|||||||||||||</td><td>1</td><td>19</td></tr>
<tr><td>miR319</td><td>XM_044485861.1</td><td>transcription factor GAMYB (LOC543161),transcript variant X2, mRNA</td><td>x|||||x|||||||||||||</td><td>1</td><td>19</td></tr>
<tr><td>miR319</td><td>XM_044485860.1</td><td>transcription factor GAMYB (LOC543161),transcript variant X1, mRNA</td><td>x|||||x|||||||||||||</td><td>1</td><td>19</td></tr>
<tr><td>miR319</td><td>XM_044594983.1</td><td>transcription factor GAMYB-like(LOC123182426), transcript variant X2, mRNA</td><td>x|||||x|||||||||||||</td><td>1</td><td>19</td></tr>
<tr><td>miR319</td><td>XM_044594981.1</td><td>transcription factor GAMYB-like(LOC123182426), transcript variant X1, mRNA</td><td>x|||||x|||||||||||||</td><td>1</td><td>19</td></tr>
<tr><td>miR319</td><td>XM_044557439.1</td><td>transcription factor GAMYB-like(LOC123137611), transcript variant X2, mRNA</td><td>x|||||x|||||||||||||</td><td>1</td><td>19</td></tr>
<tr><td>miR319</td><td>XM_044557432.1</td><td>transcription factor GAMYB-like(LOC123137611), transcript variant X1, mRNA</td><td>x|||||x|||||||||||||</td><td>1</td><td>19</td></tr>
<tr><td>miR319</td><td>XM_044483404.1</td><td>transcription factor GAMYB (LOC100873143),transcript variant X3, mRNA</td><td>x|||||x|||||||||||||</td><td>1</td><td>19</td></tr>
<tr><td>miR319</td><td>XM_044483396.1</td><td>transcription factor GAMYB (LOC100873143),transcript variant X2, mRNA</td><td>x|||||x|||||||||||||</td><td>1</td><td>19</td></tr>
<tr><td>miR319</td><td>XM_044483392.1</td><td>transcription factor GAMYB (LOC100873143),transcript variant X1, mRNA</td><td>x|||||x|||||||||||||</td><td>1</td><td>19</td></tr>
<tr><td>miR397</td><td>XM_044512550.1</td><td>cyclin-D5-1-like (LOC123091145),mRNA</td><td>||x|||||||||||||</td><td>1</td><td>16</td></tr>
<tr><td>miR397</td><td>XM_044534450.1</td><td>uncharacterized LOC123113253 (LOC123113253),mRNA</td><td>||||||||||||x|||</td><td>1</td><td>17</td></tr>
<tr><td>miR397</td><td>XM_044534389.1</td><td>uncharacterized LOC123113206 (LOC123113206),mRNA</td><td>|||||||||||||x|||</td><td>1</td><td>17</td></tr>
<tr><td>miR397</td><td>XM_044543569.1</td><td>nuclear pore complex protein NUP1-like(LOC123123121), mRNA</td><td>x||||||||||x|||||</td><td>2</td><td>17</td></tr>
<tr><td>miR397</td><td>XM_044494009.1</td><td>uncharacterized LOC123070715 (LOC123070715),mRNA</td><td>|||||||||||x||||</td><td>2</td><td>17</td></tr>
<tr><td>miR397</td><td>XM_044464928.1</td><td>uncharacterized LOC123042483 (LOC123042483),mRNA</td><td>||||||x|||||||||</td><td>2</td><td>17</td></tr>
<tr><td>miR397</td><td>XM_044464927.1</td><td>uncharacterized LOC123042482 (LOC123042482),mRNA</td><td>||||||x|||||||||</td><td>2</td><td>17</td></tr>
<tr><td>miR397</td><td>XM_044585114.1</td><td>uncharacterized LOC123167278 (LOC123167278),transcript variant X6, mRNA</td><td>x||||||||||x||x|||||</td><td>1</td><td>19</td></tr>
<tr><td>miR397</td><td>XM_044550315.1</td><td>uncharacterized LOC123130414 (LOC123130414),mRNA</td><td>|||||||||||||||||||||</td><td>1</td><td>21</td></tr>
<tr><td>miR397</td><td>XM_044560450.1</td><td>uncharacterized LOC123141269 (LOC123141269),mRNA</td><td>|||||||||||||||x|||||</td><td>1</td><td>21</td></tr>
<tr><td>miR398</td><td>XM_044588381.1</td><td>histone H2A-beta, sperm-like (LOC123170524),mRNA</td><td>||x|||||||||||x||</td><td>1</td><td>18</td></tr>
<tr><td>miR398</td><td>XM_044580832.1</td><td>late histone H2A.2.2-like (LOC123163074),mRNA</td><td>||x|||||||||||x||</td><td>1</td><td>18</td></tr>
<tr><td>miR398</td><td>XM_044503089.1</td><td>integrator complex subunit 9 homolog(LOC123080191), transcript variant X3, mRNA</td><td>x|||||||||||||||||||||</td><td>1</td><td>21</td></tr>
<tr><td>miR398</td><td>XM_044495451.1</td><td>integrator complex subunit 9 homolog(LOC123071881), mRNA</td><td>x|||||||||||||||||||||</td><td>1</td><td>21</td></tr>
<tr><td>miR398</td><td>XM_044486696.1</td><td>integrator complex subunit 9-like(LOC123062992), transcript variant X2, mRNA</td><td>x|||||||||||||||||||||</td><td>1</td><td>21</td></tr>
<tr><td>miR398</td><td>XM_044533473.1</td><td>uncharacterized LOC123112482 (LOC123112482),transcript variant X3, mRNA</td><td>x|||||||||||xx|||||x||</td><td>1</td><td>21</td></tr>
<tr><td>miR398</td><td>XM_044474634.1</td><td>superoxide dismutase [Cu-Zn] 4A(LOC123051671), transcript variant X1, mRNA</td><td>||||x||||||||xx||||||</td><td>1</td><td>21</td></tr>
<tr><td>miR398</td><td>XM_044466394.1</td><td>superoxide dismutase [Cu-Zn] 4A(LOC123043809), transcript variant X1, mRNA</td><td>||||x||||||||xx||||||</td><td>1</td><td>21</td></tr>
<tr><td>miR5384</td><td>XM_044520334.1</td><td>endo-1,4-beta-xylanase 5-like (LOC123098363),mRNA</td><td>x||||||||||||||||</td><td>1</td><td>16</td></tr>
<tr><td>miR5384</td><td>XM_044506533.1</td><td>endo-1,4-beta-xylanase 5-like (LOC123084982),transcript variant X2, mRNA</td><td>x||||||||||||||||</td><td>1</td><td>16</td></tr>
<tr><td>miR5384</td><td>XM_044585284.1</td><td>patatin-like protein 3 (LOC123167440),mRNA</td><td>|||||||||||||||</td><td>2</td><td>16</td></tr>
<tr><td>miR5384</td><td>XM_044465596.1</td><td>ATP sulfurylase 2-like (LOC123043218),mRNA</td><td>|||||||||||||||</td><td>2</td><td>16</td></tr>
<tr><td>miR5384</td><td>XM_044576115.1</td><td>protein SRG1-like (LOC123157937),mRNA</td><td>|||||||||||||||||</td><td>1</td><td>17</td></tr>
<tr><td>miR5384</td><td>XM_044578687.1</td><td>patatin-like protein 3 (LOC123160843),mRNA</td><td>||||||||||||||||</td><td>2</td><td>17</td></tr>
<tr><td>miR5384</td><td>XM_044531387.1</td><td>cellulose synthase-like proteinD4 (LOC123110776), mRNA</td><td>x||||||||||||||||</td><td>2</td><td>17</td></tr>
<tr><td>miR5384</td><td>XM_044523994.1</td><td>cellulose synthase-like proteinD4 (LOC123102592), mRNA</td><td>x||||||||||||||||</td><td>2</td><td>17</td></tr>
<tr><td>miR5384</td><td>XM_044483081.1</td><td>xyloglucan galactosyltransferaseKATAMARI1 homolog (LOC123060382), mRNA</td><td>||||||||||||||||</td><td>2</td><td>17</td></tr>
<tr><td>miR5384</td><td>XM_044588399.1</td><td>protein SRG1-like (LOC123170539),mRNA</td><td>|||||x|||||||||||</td><td>1</td><td>17</td></tr>
<tr><td>miR5384</td><td>XM_044587369.1</td><td>putative clathrin assembly proteinAt5g57200 (LOC123169497), mRNA</td><td>|||x|||||||||||||</td><td>1</td><td>17</td></tr>
<tr><td>miR5384</td><td>XM_044579919.1</td><td>putative clathrin assembly proteinAt5g57200 (LOC123162123), mRNA</td><td>|||x|||||||||||||</td><td>1</td><td>17</td></tr>
<tr><td>miR5384</td><td>XM_044566363.1</td><td>expansin-like A4 (LOC123147128),mRNA</td><td>|||||||||||x|||||</td><td>1</td><td>17</td></tr>
<tr><td>miR5384</td><td>XM_044562287.1</td><td>uncharacterized LOC123143370 (LOC123143370),mRNA</td><td>||||||||||||||x||</td><td>1</td><td>17</td></tr>
</table>

<table>
<tr><td>miR5384</td><td>XM_044562106.1</td><td>protein trichome birefringence-like12 (LOC123143252), mRNA</td><td>||x||||||||||||||</td><td>1</td><td>17</td></tr>
<tr><td>miR5384</td><td>XM_044555076.1</td><td>uncharacterized LOC123135847 (LOC123135847),mRNA</td><td>||||||||||||||x||</td><td>1</td><td>17</td></tr>
<tr><td>miR5384</td><td>XM_044554860.1</td><td>protein trichome birefringence-like12 (LOC123135683), mRNA</td><td>||x||||||||||||||</td><td>1</td><td>17</td></tr>
<tr><td>miR5384</td><td>XM_044551481.1</td><td>protein trichome birefringence-like12 (LOC123131800), mRNA</td><td>||x||||||||||||||</td><td>1</td><td>17</td></tr>
<tr><td>miR5384</td><td>XM_044550141.1</td><td>uncharacterized LOC123130189 (LOC123130189),mRNA</td><td>||||||||||||||x||</td><td>1</td><td>17</td></tr>
<tr><td>miR5384</td><td>XM_044538768.1</td><td>uncharacterized LOC123119094 (LOC123119094),mRNA</td><td>|||||||||||||x||</td><td>1</td><td>17</td></tr>
<tr><td>miR5384</td><td>XM_044536809.1</td><td>uncharacterized LOC123115702 (LOC123115702),transcript variant X2, mRNA</td><td>|||||||||||||x|||</td><td>1</td><td>17</td></tr>
<tr><td>miR5384</td><td>XM_044536574.1</td><td>uncharacterized LOC123115415 (LOC123115415),transcript variant X2, mRNA</td><td>||||||||||||||x||</td><td>1</td><td>17</td></tr>
<tr><td>miR5384</td><td>XM_044530974.1</td><td>uncharacterized LOC123110454 (LOC123110454),transcript variant X2, mRNA</td><td>|||||||||||||x|||</td><td>1</td><td>17</td></tr>
<tr><td>miR5384</td><td>XM_044523229.1</td><td>uncharacterized LOC123101979 (LOC123101979),mRNA</td><td>|||||||||||||x||</td><td>1</td><td>17</td></tr>
<tr><td>miR5384</td><td>XM_044516912.1</td><td>uncharacterized LOC123095084 (LOC123095084),transcript variant X2, mRNA</td><td>x|||||||x|||||||||</td><td>1</td><td>17</td></tr>
<tr><td>miR5384</td><td>XM_044505510.1</td><td>haloacid dehalogenase-like hydrolasedomain-containing protein Sgpp (LOC123083473), mRNA</td><td>||x||||||||||||||</td><td>1</td><td>17</td></tr>
<tr><td>miR5384</td><td>XM_044499388.1</td><td>peroxisomal fatty acid beta-oxidationmultifunctional protein-like (LOC123077174), mRNA</td><td>||||||||x||||||||</td><td>1</td><td>17</td></tr>
<tr><td>miR5384</td><td>XM_044491259.1</td><td>peroxisomal fatty acid beta-oxidationmultifunctional protein-like (LOC123068651), mRNA</td><td>||||||||x||||||||</td><td>1</td><td>17</td></tr>
<tr><td>miR5384</td><td>XM_044482740.1</td><td>peroxisomal fatty acid beta-oxidationmultifunctional protein-like (LOC123060164), mRNA</td><td>||||||||x||||||||</td><td>1</td><td>17</td></tr>
<tr><td>miR5384</td><td>XM_044488569.1</td><td>probable aquaporin TIP4-2 (LOC123065226),mRNA</td><td>||||||||||||||x|||</td><td>1</td><td>18</td></tr>
<tr><td>miR5384</td><td>XM_044480987.1</td><td>probable aquaporin TIP4-2 (LOC123058173),mRNA</td><td>||||||||||||||x|||</td><td>1</td><td>18</td></tr>
<tr><td>miR5384</td><td>XM_044462332.1</td><td>COBRA-like protein 7 (LOC123038984),mRNA</td><td>|||||x||||||||||||</td><td>1</td><td>18</td></tr>
<tr><td>miR5384</td><td>XM_044596739.1</td><td>COBRA-like protein 7 (LOC123184655),mRNA</td><td>|||||x||||||||||||</td><td>1</td><td>18</td></tr>
<tr><td>miR5384</td><td>XM_044595983.1</td><td>3-ketoacyl-CoA synthase 11-like(LOC123183220), mRNA</td><td>x||||||||||||||x|||</td><td>1</td><td>18</td></tr>
<tr><td>miR5384</td><td>XM_044566960.1</td><td>3-ketoacyl-CoA synthase 11-like(LOC123147685), mRNA</td><td>x||||||||||||||x|||</td><td>1</td><td>18</td></tr>
<tr><td>miR5384</td><td>XM_044566152.1</td><td>3-ketoacyl-CoA synthase 4-like (LOC123146880),mRNA</td><td>x||||||||||||||x|||</td><td>1</td><td>18</td></tr>
<tr><td>miR5384</td><td>XM_044492022.1</td><td>3-ketoacyl-CoA synthase 11-like(LOC123069222), mRNA</td><td>x||||||||||||||x|||</td><td>1</td><td>18</td></tr>
<tr><td>miR5384</td><td>XM_044588862.1</td><td>phospholipase D alpha 2-like (LOC123171299),mRNA</td><td>||||x||||||||||||</td><td>2</td><td>18</td></tr>
<tr><td>miR5384</td><td>XM_044586766.1</td><td>probable L-type lectin-domain containingreceptor kinase S.5 (LOC123168906), mRNA</td><td>||||||||||||||x||</td><td>2</td><td>18</td></tr>
<tr><td>miR5384</td><td>XM_044576583.1</td><td>BTB/POZ and MATH domain-containingprotein 2-like (LOC123158684), mRNA</td><td>|||x||||||||||||</td><td>2</td><td>18</td></tr>
<tr><td>miR5384</td><td>XM_044573889.1</td><td>phospholipase D alpha 2-like (LOC123155744),mRNA</td><td>||||x||||||||||||</td><td>2</td><td>18</td></tr>
<tr><td>miR5384</td><td>XM_044571396.1</td><td>probable L-type lectin-domain containingreceptor kinase S.5 (LOC123151735), mRNA</td><td>||||||||||||||x||</td><td>2</td><td>18</td></tr>
<tr><td>miR5384</td><td>XM_044571346.1</td><td>probable L-type lectin-domain containingreceptor kinase S.5 (LOC123151679), mRNA</td><td>||||||||||||||x||</td><td>2</td><td>18</td></tr>
<tr><td>miR5384</td><td>XM_044566355.1</td><td>phospholipase D alpha 2-like (LOC123147117),mRNA</td><td>||||x||||||||||||</td><td>2</td><td>18</td></tr>
<tr><td>miR5384</td><td>XM_044562105.1</td><td>autophagy protein 5-like (LOC123143250),mRNA</td><td>||||x||||||||||||</td><td>2</td><td>18</td></tr>
<tr><td>miR5384</td><td>XM_044547687.1</td><td>uncharacterized LOC123127832 (LOC123127832),mRNA</td><td>||||||||||||||x||</td><td>2</td><td>18</td></tr>
<tr><td>miR5384</td><td>XM_044526353.1</td><td>homeobox-leucine zipper proteinROC6-like (LOC123104487), transcript variant X3, mRNA</td><td>|||||||||||x|||||</td><td>2</td><td>18</td></tr>
<tr><td>miR5384</td><td>XM_044516454.1</td><td>probable CCR4-associated factor1 homolog 11 (LOC123094448), mRNA</td><td>||||||||||x||||||</td><td>2</td><td>18</td></tr>
<tr><td>miR5384</td><td>XM_044516453.1</td><td>probable CCR4-associated factor1 homolog 11 (LOC123094447), mRNA</td><td>||||||||||x||||||</td><td>2</td><td>18</td></tr>
<tr><td>miR5384</td><td>XM_044516451.1</td><td>probable CCR4-associated factor1 homolog 11 (LOC123094443), mRNA</td><td>||||||||||x||||||</td><td>2</td><td>18</td></tr>
<tr><td>miR5384</td><td>XM_044516450.1</td><td>probable CCR4-associated factor1 homolog 11 (LOC123094442), mRNA</td><td>||||||||||x||||||</td><td>2</td><td>18</td></tr>
<tr><td>miR5384</td><td>XM_044516449.1</td><td>probable CCR4-associated factor1 homolog 11 (LOC123094441), mRNA</td><td>||||||||||x||||||</td><td>2</td><td>18</td></tr>
<tr><td>miR5384</td><td>XM_044516448.1</td><td>probable CCR4-associated factor1 homolog 11 (LOC123094440), mRNA</td><td>||||||||||x||||||</td><td>2</td><td>18</td></tr>
<tr><td>miR5384</td><td>XM_044516447.1</td><td>probable CCR4-associated factor1 homolog 11 (LOC123094439), mRNA</td><td>||||||||||x||||||</td><td>2</td><td>18</td></tr>
<tr><td>miR5384</td><td>XM_044516446.1</td><td>probable CCR4-associated factor1 homolog 11 (LOC123094438), mRNA</td><td>||||||||||x||||||</td><td>2</td><td>18</td></tr>
<tr><td>miR5384</td><td>XM_044516445.1</td><td>probable CCR4-associated factor1 homolog 11 (LOC123094437), mRNA</td><td>||||||||||x||||||</td><td>2</td><td>18</td></tr>
<tr><td>miR5384</td><td>XM_044509825.1</td><td>protein DETOXIFICATION 54-like (LOC123087729),mRNA</td><td>|||||||||||||x|||</td><td>2</td><td>18</td></tr>
<tr><td>miR5384</td><td>XM_044504655.1</td><td>probable CCR4-associated factor1 homolog 11 (LOC123082292), mRNA</td><td>||||||||||x||||||</td><td>2</td><td>18</td></tr>
<tr><td>miR5384</td><td>XM_044502624.1</td><td>GABA transporter 1-like (LOC123079810),mRNA</td><td>||||||||||x||||||</td><td>2</td><td>18</td></tr>
<tr><td>miR5384</td><td>XM_044495046.1</td><td>GABA transporter 1-like (LOC123071474),mRNA</td><td>||||||||||x||||||</td><td>2</td><td>18</td></tr>
<tr><td>miR5384</td><td>XM_044488055.1</td><td>putative glutamine amidotransferaseGAT1_2.1 (LOC123064607), mRNA</td><td>|||||||x|||||||||</td><td>2</td><td>18</td></tr>
<tr><td>miR5384</td><td>XM_044486264.1</td><td>GABA transporter 1-like (LOC123062653),mRNA</td><td>||||||||||x||||||</td><td>2</td><td>18</td></tr>
<tr><td>miR5384</td><td>XM_044482103.1</td><td>probable Histone-lysine N-methyltransferaseATXR5 (LOC123059561), mRNA</td><td>|||x||||||||||||</td><td>2</td><td>18</td></tr>
<tr><td>miR5384</td><td>XM_044592093.1</td><td>glycine-rich cell wall structuralprotein-like (LOC123180129), transcript variant X3, mRNA</td><td>|||||||x|||||||||</td><td>2</td><td>18</td></tr>
<tr><td>miR5384</td><td>XM_044591931.1</td><td>phospholipase D alpha 2-like (LOC123179989),mRNA</td><td>||||x||||||||||||</td><td>2</td><td>18</td></tr>
<tr><td>miR5384</td><td>XM_044591622.1</td><td>uncharacterized LOC123179749 (LOC123179749),mRNA</td><td>||||||||||x||||||</td><td>2</td><td>18</td></tr>
<tr><td>miR5384</td><td>XM_044592002.1</td><td>nuclear transcription factor Y subunitC-3-like (LOC123180040), mRNA</td><td>||||||||||||||x||</td><td>2</td><td>18</td></tr>
</table>

<table>
<tr><td>miR5384</td><td>XM_044590544.1</td><td>probable L-type lectin-domain containingreceptor kinase S.5 (LOC123176243), mRNA</td><td>||||||||||||||x||</td><td>2</td><td>18</td></tr>
<tr><td>miR5384</td><td>XM_044590460.1</td><td>probable L-type lectin-domain containingreceptor kinase S.5 (LOC123176067), mRNA</td><td>||||||||||||||x||</td><td>2</td><td>18</td></tr>
<tr><td>miR5384</td><td>XM_044550663.1</td><td>aspartyl protease family proteinAt5g10770-like (LOC123130872), mRNA</td><td>x|||x|||||||||||||||</td><td>1</td><td>19</td></tr>
<tr><td>miR5384</td><td>XM_044543288.1</td><td>cytochrome P450 81Q32-like (LOC123122911),mRNA</td><td>|||||||x||||||||||</td><td>2</td><td>19</td></tr>
<tr><td>miR5384</td><td>XM_044534613.1</td><td>cytochrome P450 81Q32-like (LOC123113393),mRNA</td><td>|||||||x||||||||||</td><td>2</td><td>19</td></tr>
<tr><td>miR5384</td><td>XM_044534612.1</td><td>cytochrome P450 81Q32-like (LOC123113392),mRNA</td><td>|||||||x||||||||||</td><td>2</td><td>19</td></tr>
<tr><td>miR5384</td><td>XM_044527095.1</td><td>cytochrome P450 81Q32-like (LOC123105103),mRNA</td><td>|||||||x||||||||||</td><td>2</td><td>19</td></tr>
<tr><td>miR5384</td><td>XM_044479899.1</td><td>aspartyl protease family proteinAt5g10770-like (LOC123056215), mRNA</td><td>||x|||||||||||||||</td><td>2</td><td>19</td></tr>
<tr><td>miR5384</td><td>XM_044581457.1</td><td>pentatricopeptide repeat-containingprotein At1g80150, mitochondrial-like (LOC123164034), mRNA</td><td>||||||||x||x|||||||</td><td>1</td><td>19</td></tr>
<tr><td>miR5384</td><td>XM_044574457.1</td><td>pentatricopeptide repeat-containingprotein At1g80150, mitochondrial-like (LOC123156317), transcript</td><td>||||||||x||x|||||||</td><td>1</td><td>19</td></tr>
<tr><td>miR5384</td><td>XM_044570782.1</td><td>pentatricopeptide repeat-containingprotein At1g80150, mitochondrial-like (LOC123150985), mRNA</td><td>||||||||x||x|||||||</td><td>1</td><td>19</td></tr>
<tr><td>miR5384</td><td>XM_044548118.1</td><td>uncharacterized LOC123128183 (LOC123128183),mRNA</td><td>||||||x|||||||x||||</td><td>1</td><td>19</td></tr>
<tr><td>miR5384</td><td>XM_044544958.1</td><td>CASP-like protein 1B2 (LOC123124324),mRNA</td><td>||||||||x|||||x||||</td><td>1</td><td>19</td></tr>
<tr><td>miR5384</td><td>XM_044540613.1</td><td>probable carboxylesterase 12 (LOC123120609),mRNA</td><td>|||x|||||||||||x|||</td><td>1</td><td>19</td></tr>
<tr><td>miR5384</td><td>XM_044533171.1</td><td>probable carboxylesterase 12 (LOC123112232),mRNA</td><td>|||x|||||||||||x|||</td><td>1</td><td>19</td></tr>
<tr><td>miR5384</td><td>XM_044532368.1</td><td>SKP1-like protein 1A (LOC123111558),mRNA</td><td>||x||x|||||||||||||</td><td>1</td><td>19</td></tr>
<tr><td>miR5384</td><td>XM_044525718.1</td><td>probable carboxylesterase 12 (LOC123104005),mRNA</td><td>|||x|||||||||||x|||</td><td>1</td><td>19</td></tr>
<tr><td>miR5384</td><td>XM_044518193.1</td><td>protein SMAX1-LIKE 3-like (LOC123096448),mRNA</td><td>||x||||||||||||x|||</td><td>1</td><td>19</td></tr>
<tr><td>miR5384</td><td>XM_044513014.1</td><td>protein SMAX1-LIKE 3-like (LOC123091485),mRNA</td><td>||x||||||||||||x|||</td><td>1</td><td>19</td></tr>
<tr><td>miR5384</td><td>XM_044508271.1</td><td>protein SMAX1-LIKE 3-like (LOC123086511),mRNA</td><td>||x||||||||||||x|||</td><td>1</td><td>19</td></tr>
<tr><td>miR5384</td><td>XM_044599587.1</td><td>uncharacterized LOC123187669 (LOC123187669),mRNA</td><td>||||||||||||x|x||||</td><td>1</td><td>19</td></tr>
<tr><td>miR5384</td><td>XM_044598736.1</td><td>subtilisin-like protease (LOC123186992),mRNA</td><td>x|||||||||||x||x||||</td><td>1</td><td>19</td></tr>
<tr><td>miR5384</td><td>XM_044596013.1</td><td>probable amidase At4g34880 (LOC123183245),mRNA</td><td>|||||x||||||||x||||</td><td>1</td><td>19</td></tr>
<tr><td>miR5384</td><td>XM_044592444.1</td><td>uncharacterized LOC123180398 (LOC123180398),mRNA</td><td>||||||||x|||x||||||</td><td>1</td><td>19</td></tr>
<tr><td>miR5384</td><td>XM_044523526.1</td><td>CASP-like protein 1B2 (LOC123102233),mRNA</td><td>||||||||x|||||x||||||</td><td>1</td><td>21</td></tr>
<tr><td>miR9657</td><td>XM_044565725.1</td><td>uncharacterized LOC123146139 (LOC123146139),transcript variant X1, mRNA</td><td>|||||||||||||x||</td><td>1</td><td>16</td></tr>
<tr><td>miR9657</td><td>XM_044532826.1</td><td>uncharacterized LOC123111942 (LOC123111942),transcript variant X3, mRNA</td><td>||||x|||||||||||</td><td>1</td><td>16</td></tr>
<tr><td>miR9657</td><td>XM_044522883.1</td><td>serine/threonine-protein phosphatase7 long form homolog (LOC123101426), mRNA</td><td>||||x|||||||||||</td><td>1</td><td>16</td></tr>
<tr><td>miR9657</td><td>XM_044564033.1</td><td>serine/threonine-protein phosphatase7 long form homolog (LOC123144790), mRNA</td><td>||||x|||||||||||</td><td>1</td><td>16</td></tr>
<tr><td>miR9657</td><td>XM_044586773.1</td><td>uncharacterized LOC123168910 (LOC123168910),transcript variant X4, mRNA</td><td>x||x|||||||||x|||||</td><td>1</td><td>18</td></tr>
<tr><td>miR9657</td><td>XM_044495358.1</td><td>signal recognition particle subunitSRP72-like (LOC123071773), mRNA</td><td>x|||x|||||||x|||x|||||</td><td>1</td><td>21</td></tr>
<tr><td>miR9670</td><td>XM_044578391.1</td><td>disease resistance protein RGA2-like(LOC123160587), transcript variant X2, mRNA</td><td>||||||||x||x||||||</td><td>1</td><td>18</td></tr>
<tr><td>miR9670</td><td>XM_044590257.1</td><td>disease resistance protein RGA5-like(LOC123175631), mRNA</td><td>x|||||x|||||||x||||</td><td>1</td><td>18</td></tr>
<tr><td>miR9670</td><td>XM_044570553.1</td><td>G-type lectin S-receptor-like serine/threonine-proteinkinase SD2-5 (LOC123150717), transcript</td><td>|||||||xx|||||||||||</td><td>1</td><td>20</td></tr>
<tr><td>miR9670</td><td>XM_044598261.1</td><td>mavicyanin-like (LOC123186502),mRNA</td><td>||||||x||||||||||x||</td><td>2</td><td>21</td></tr>
<tr><td>miR9773</td><td>XM_044477988.1</td><td>uncharacterized LOC123054258 (LOC123054258),mRNA</td><td>x|||||||||||||x||</td><td>2</td><td>17</td></tr>
<tr><td>miR9773</td><td>XM_044514387.1</td><td>uncharacterized LOC123092586 (LOC123092586),transcript variant X2, mRNA</td><td>x|||||||||||x|x||||</td><td>1</td><td>18</td></tr>
<tr><td>miR9773</td><td>XM_044554868.1</td><td>putative F-box/LRR-repeat/kelch-repeatprotein At1g11620 (LOC123135691), transcript variant</td><td>x|||x|||||||||x|||||</td><td>1</td><td>19</td></tr>
<tr><td>miR9773</td><td>XM_044506190.1</td><td>uncharacterized LOC123084700 (LOC123084700),mRNA</td><td>||||||||||x|x|||||</td><td>2</td><td>19</td></tr>
<tr><td>miR9773</td><td>XM_044557206.1</td><td>enhancer of rudimentary homolog(LOC123137446), transcript variant X2, mRNA</td><td>||||||||x||||x|||||</td><td>1</td><td>20</td></tr>
<tr><td>miR9773</td><td>XM_044491886.1</td><td>uncharacterized LOC123069123 (LOC123069123),transcript variant X3, mRNA</td><td>|||x|x||||||||||||||</td><td>1</td><td>20</td></tr>
<tr><td>miR9773</td><td>XM_044601695.1</td><td>MADS-box transcription factor 26-like(LOC123189307), transcript variant X1, mRNA</td><td>x|||||||||||x|x||x|||||||</td><td>1</td><td>24</td></tr>
<tr><td>miR9773</td><td>XM_044490241.1</td><td>uncharacterized LOC123067455 (LOC123067455),mRNA</td><td>x|||x||||||x|||||||x||||</td><td>1</td><td>24</td></tr>
<tr><td>miR9774</td><td>XM_044598808.1</td><td>glyoxylate/hydroxypyruvate reductaseHPR3-like (LOC123187045), mRNA</td><td>x|||||||||||x|||||</td><td>1</td><td>17</td></tr>
<tr><td>miR9774</td><td>XM_044472107.1</td><td>photosystem II protein D1-like (LOC123049085),partial mRNA</td><td>||||||||||||||||||||||</td><td>1</td><td>22</td></tr>
</table>

Figure S1

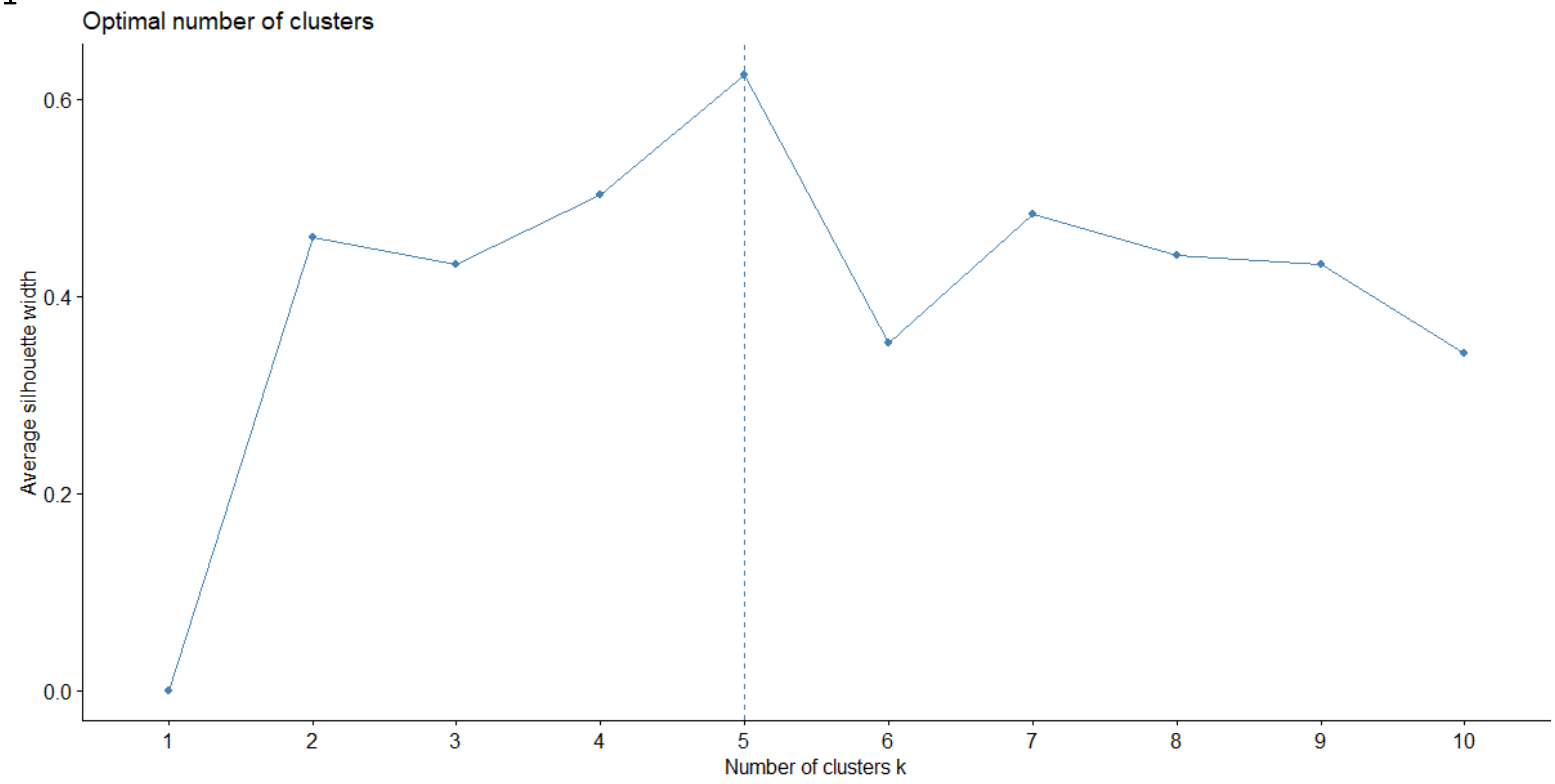


**Figure S1. The optimum number of clusters determined by the average silhouette method**. A distinct peak observed at five indicates the optimum number of clusters. Additionally, two local maxima at two and seven clusters were also observed.

Figure S2

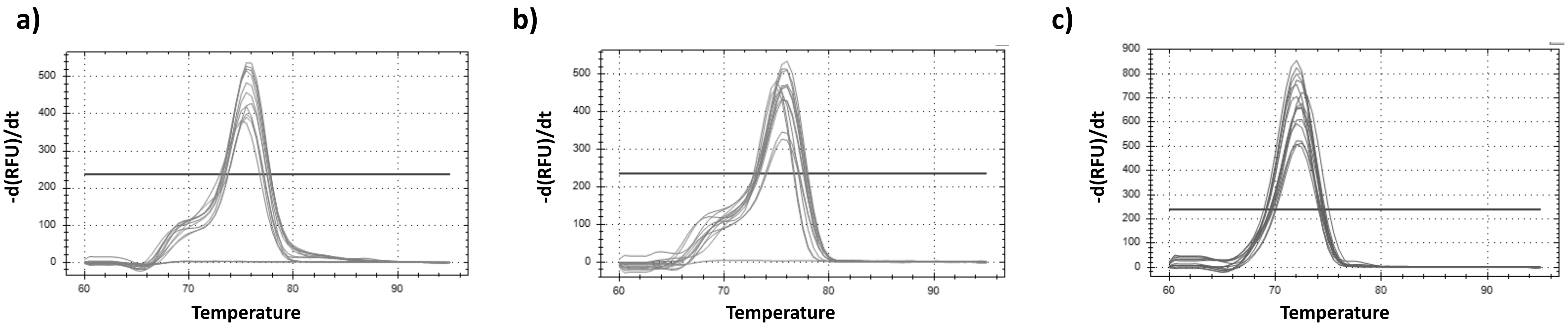


**Figure S2. The dissociation curves for the qPCR products amplified with pTR and a) Tae397F, b) Tae398F, and c) Tae9670F**. Each curve exhibited a single peak, supporting the specificity of the amplification.
